# Assessment of tumor suppressor promoter methylation in healthy individuals

**DOI:** 10.1186/s13148-020-00920-7

**Published:** 2020-08-28

**Authors:** Deepak B. Poduval, Elisabet Ognedal, Zuzana Sichmanova, Eivind Valen, Gjertrud T. Iversen, Laura Minsaas, Per E. Lønning, Stian Knappskog

**Affiliations:** 1 K.G. Jebsen Center for Genome Directed Cancer Therapy, Department of Clinical Science, University of Bergen, Bergen, Norway; 2 Department of Oncology, Haukeland University Hospital, Bergen, Norway; 3 Present address: Department of Medical Genetics, Haukeland University Hospital, Bergen, Norway; 4 Computational Biology Unit, Department of Informatics, University of Bergen, Bergen, Norway; 5 Sars International Centre for Marine Molecular Biology, University of Bergen, Bergen, Norway

**Keywords:** Methylation, Epimutations, Cancer risk, Promoter, Massive parallel sequencing

## Abstract

**Background:**

The number of tumor suppressor genes for which germline mutations have been linked to cancer risk is steadily increasing. However, while recent reports have linked constitutional normal tissue promoter methylation of *BRCA1* and *MLH1* to ovarian and colon cancer risk, the role of epigenetic alterations as cancer risk factors remains largely unknown, presenting an important area for future research. Currently, we lack fast and sensitive methods for assessment of promoter methylation status across known tumor suppressor genes.

**Results:**

In this paper, we present a novel NGS-based approach assessing promoter methylation status across a large panel of defined tumor suppressor genes to base-pair resolution. The method omits the limitations related to commonly used array-approaches. Our panel includes 565 target regions covering the promoters of 283 defined tumor suppressors, selected by pre-specified criteria, and was applied for rapid targeted methylation-specific NGS. The feasibility of the method was assessed by analyzing normal tissue DNA (white blood cells, WBC) samples from 34 healthy postmenopausal women and by performing preliminary assessment of the methylation landscape of tumor suppressors in these individuals. The mean target coverage was 189.6x providing a sensitivity of 0.53%, sufficient for promoter methylation assessment of low-level methylated genes like *BRCA1*. Within this limited test-set, we detected 206 regions located in the promoters of 149 genes to be differentially methylated (*hyper*- or *hypo*-) at > 99% confidence level. Seven target regions in gene promoters (*CIITA*, *RASSF1*, *CHN1*, *PDCD1LG2*, *GSTP1*, *XPA*, and *ZNF668*) were found to be *hyper*-methylated in a minority of individuals, with a > 20 percent point difference in mean methylation across the region between individuals. In an exploratory hierarchical clustering analysis, we found that the individuals analyzed may be grouped into two main groups based on their WBC methylation profile across the 283 tumor suppressor gene promoters.

**Conclusions:**

Methylation-specific NGS of our tumor suppressor panel, with detailed assessment of differential methylation in healthy individuals, presents a feasible method for identification of novel epigenetic risk factors for cancer.

## Introduction

The number of tumor suppressor genes for which germline mutations are linked to elevated cancer risk is steadily increasing [1–3]. Mutations across different genes present a continuum of penetrance, ranging from moderately to massively elevated risk of different cancer forms. Further, while mutations in some genes (so far) are restricted to increased risk of a single, or a few cancer forms, mutations in other genes may increase the risk of multiple different types of cancer [4, 5].

Some of the best described “classical” high penetrance genes include *BRCA1/2*, for which germline mutations are associated with an elevated risk of ovarian and breast cancer [6], *MLH1/MSH2* linked to colorectal cancer [7], *CDKN2A* and *RB1*, associated with melanoma and retinoblastoma, respectively [8–10], as well as *TP53*, associated with the Li-Fraumeni syndrome with an elevated risk for multiple cancer forms [11]. However, the list of genes for which germline mutations are ascertained to confer cancer risk is continuously increasing due to application of massive parallel sequencing [12, 13]. Still, for many families with multiple cases of a specific tumor form (like breast, ovary, or melanomas), no pathogenic germline gene variant has been identified.

Epigenetic gene inactivation may occur through different mechanisms [14, 15]. So far, promoter methylation is the best studied of all the epigenetic modifications, and such methylation is well established as a mechanism of inactivation of tumor suppressor genes. While many germline mutations affecting tumor suppressor genes are well studied as cancer risk factors, knowledge regarding constitutional epigenetic inactivation [16] as a potential cancer risk factor remains limited. Somatic promotor methylation in tumor suppressor genes is a common event in cancer [17], but the role of aberrant epigenetic events, or constitutional promoter methylation of tumor suppressor genes in normal cells as potential cancer risk factors, remains largely unexplored. While mosaic methylation of the *MLH1* gene in normal leukocytes has been observed in colorectal cancer patients [18, 19] and a haplotype leading to secondary constitutional methylation in the *MGM2* promoter [20] has been found in a cancer-prone family [21], in general, data on normal tissue methylation patterns and cancer risk are scarce [22].

Recently, in a large study, we reported low-grade mosaic (< 10% of alleles) normal tissue *BRCA1* promoter methylation to confer a significantly increased risk of high-grade serous ovarian cancer (HGSOC) [23]. In our study, we found > 4% of healthy adult females in a Caucasian population to harbor mosaic *BRCA1* promoter methylation in their normal white blood cells (WBC). Individuals carrying such methylation had a 2-3 fold increased risk of HGSOC. Importantly, WBC *BRCA1* promoter methylation was strongly associated with corresponding methylation in other normal tissues, and, in HGSOC patients, also associated with methylation in the tumor. Taken together, this indicated that methylated normal cells in the ovary may act as tumor precursors.

Based on these results and the findings of others [19, 24–29], we hypothesized that additional tumor suppressors could be hyper-methylated in normal cells, thereby causing an elevated risk for certain cancer forms within subgroups of healthy individuals in the general population [30].

To explore such a hypothesis, there is a need for improved methodologies. Although methylation status may be analyzed by conventional arrays, such assessments are limited to the selection of CpGs covered by the array probes. These selected CpGs may not necessarily represent all the CpGs crucial for gene silencing [23]. An alternative is methylation-specific whole genome sequencing, but this remains prohibitively costly. In the present study, we aimed to establish, and provide proof-of-concept for, a novel strategy assessing the full CpG spectrum across promoter areas of tumor suppressor genes. The assay applies methylation-specific massive parallel sequencing of the promoter areas of a panel of 283 tumor suppressor genes. We show the feasibility of the method by depicting promoter methylation variation across the promoter panel in a set of white blood cell (WBC) DNA obtained from 34 healthy individuals. Further, by performing an exploratory hierarchical clustering, our findings indicate that the profiles of normal cell promoter methylation of tumor suppressor genes fall into two main clusters defined by differences in genes regulating key biological pathways.

## Results

### Methylation specific sequencing

**Fig. 1:**
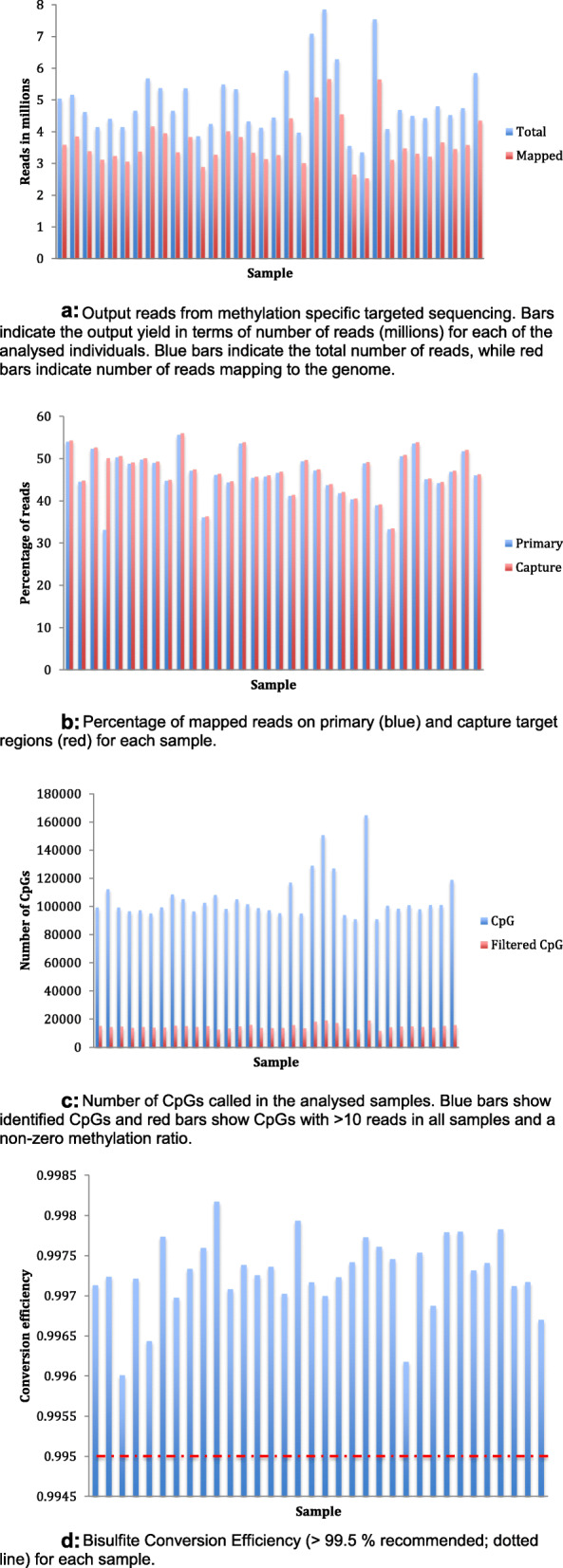
**a** Output reads from methylation specific targeted sequencing. Bars indicate the output yield in terms of number of reads (millions) for each of the analyzed individuals. Blue bars indicate the total number of reads, while red bars indicate number of reads mapping to the genome. **b** Percentage of mapped reads on primary (blue) and capture target regions (red) for each sample. **c** Number of CpGs called in the analyzed samples. Blue bars show identified CpGs and red bars show CpGs with > 10 reads in all samples and a non-zero methylation ratio. **d** Bisulfite conversion efficiency (> 99.5% recommended; dotted line) for each sample

We analyzed WBC DNA from 34 healthy individuals. After bisulfite conversion of the DNA, we performed methylation-specific sequencing of 565 capture regions representing 356 target regions from 283 tumor suppressor gene promoters (the full list of genes and regions is presented as Supplementary Table S1). Sequencing was performed on an Illumina MiSeq, running 8 samples per run. Regarding average values per sample, we obtained 4.95 × 10^6^ reads (range 3.36-7.85 × 10^6^) (Fig. 1a; for details per sample see Table 1). Subsequent to quality filtering, 88% of the reads, were retained. Thus, after filtering, 4.30 × 10^6^ reads were attempted mapped to the genome, yielding 4.08 × 10^6^ mapped single reads. Out of these, 3.6 × 10^6^ reads mapped with properly paired reads for each sample (average values; Fig. 1a). These reads led to a mean primary target coverage of 189.6x (114.8x-269.5x) and a mean capture target coverage of 199.4x (120.7x-283.4x). Every sample had almost equal percentage of reads mapped to capture targets and primary targets (Fig. 1b).

**Table 1:** Summary of samples and analyses

Sample	Input reads	% reads post QC	% reads mapped	Reads (paired and clipped)	% reads on target (primary)	% reads on target (capture)	Coverage on target (primary)	Coverage on target (capture)
**10046_S2**	5043578	90.39	78.71	3588074	54.01	54.31	209.99	220.84
**10071_S7**	5167738	89.08	83.62	3849392	44.54	44.81	186.40	196.12
**10077_S3**	4622790	90.13	81.18	3382228	52.35	52.66	198.17	208.34
**10078_S8**	4148304	89.91	83.59	3117970	33.13	50.13	175.26	184.27
**10081_S4**	4408742	89.53	81.99	3236368	50.31	50.60	186.37	195.79
**10082_S5**	4146244	88.90	82.83	3053058	48.82	49.10	171.00	179.62
**10086_S6**	4665150	89.39	80.91	3373894	49.76	50.06	186.09	195.68
**10088_S2**	5683572	88.12	83.26	4169990	49.01	49.29	219.04	230.48
**10097_S3**	5372752	87.46	84.09	3951544	44.74	45.00	195.13	205.17
**10107_S1**	4659718	89.67	80.24	3352898	55.68	56.00	213.95	224.78
**10110_S6**	5369964	86.87	82.21	3835038	47.17	47.45	199.76	210.11
**10113_S7**	3862862	89.44	83.54	2886124	36.11	36.31	120.48	126.52
**10117_S5**	4243470	89.93	85.80	3274194	46.15	46.42	169.10	177.78
**10126_S7**	5490894	87.68	83.36	4013294	44.39	44.65	198.79	209.00
**10131_S1**	5338282	87.00	82.62	3836988	53.58	53.88	232.54	244.35
**10146_S6**	4326882	90.13	85.57	3337118	45.47	45.73	168.34	177.00
**10149_S7**	4129130	90.01	84.46	3139126	45.78	46.04	159.55	167.77
**10155_S5**	4450890	86.31	84.99	3264796	46.65	46.91	170.60	179.37
**20011_S4**	5923516	87.27	85.49	4419628	41.19	41.42	203.82	214.20
**20019_S1**	3972578	89.46	84.62	3007020	49.39	49.68	163.80	172.27
**20022_S4**	7091616	88.48	81.02	5083584	47.19	47.47	257.88	271.25
**20023_S1**	7854298	88.16	81.78	5662728	43.72	43.98	269.47	283.35
**20024_S5**	6284900	88.04	82.23	4549698	41.85	42.09	215.05	225.92
**20062_S2**	3554848	88.54	84.13	2648098	40.36	40.54	127.76	133.78
**20068_S3**	3355298	89.51	84.38	2534292	48.89	49.18	138.43	145.54
**20078_S2**	7541786	88.33	84.85	5651984	38.94	39.16	246.67	259.20
**20088_S4**	4083996	90.28	84.33	3109332	33.27	33.46	114.79	120.70
**20092_S3**	4686060	89.01	83.38	3477852	50.58	50.88	196.38	206.39
**20098_S1**	4501632	88.21	83.23	3305210	53.58	53.89	197.69	207.78
**20106_S2**	4430250	86.96	83.43	3214408	45.12	45.32	173.69	181.84
**20117_S4**	4802994	88.70	86.01	3664364	44.22	44.48	178.18	187.37
**20119_S5**	4525080	89.19	85.57	3453670	46.90	47.16	180.35	189.59
**20122_S6**	4741150	88.79	85.18	3585870	51.76	52.07	204.62	215.16
**20160_S3**	5848296	88.30	84.30	4353508	46.02	46.28	220.04	231.29

The overall number of informative CpGs identified for each sample were on average 1.0 × 10^5^ (range 0.9 × 10^5^ to 1.6 × 10^5^). Restricting the CpGs to those with a methylation ratio > 0, and more than 10 reads in coverage, the number was reduced to 1.5 × 10^4^ (range 1.1 × 10^4^-1.9 × 10^4^; Fig. 1c).

We defined the sensitivity of our strategy as 1/x, where *x* = sequencing depth at any given CpG. With the average primary target depth being 189.6x, the sensitivity was 0.53%. In theory, the fragility of this sensitivity estimate lies in that, for some samples, the results may depend on a single read, rendering them more sensitive to artifacts such as inadequate bisulfite conversion. However, assessing the bisulfite conversion rate (C to T) of the internal Lambda DNA control (see the “Methods” section), we found the conversion efficiency to be on average 99.7% (range 99.6-99.8%) across the analyzed samples (Fig. 1d). This indicates a rate of technical artifacts (falsely retained C’s instead of T’s) to be lower than 0.2-0.4%, thus approaching the error rate in the sequencing per se (Q30 threshold).

Reproducibility was assessed in a separate standard sample (pooled DNA from 5 healthy donors) that was run in 6 parallels per run over 2 independent runs. In a selection of 12 out of the 565 regions, we found the mean coefficient of variation to be 7.1% (median 4.4%; Supplementary Table S2). As such, the technical variability in this standard sample was considerably lower than the detected biological variation (see below) in our study set of 34. Variability was considerably lower when assessing all CpGs in a region than when limiting analyses to randomized selections of CpGs within the regions (e.g., for *PRDM2*, the coefficient of variation was 1.5% when considering all CpGs while it was on average 4.7% when assessing randomized selections of 5 CpGs within the region).

### Methylation landscape of tumor suppressors

**Fig. 2:**
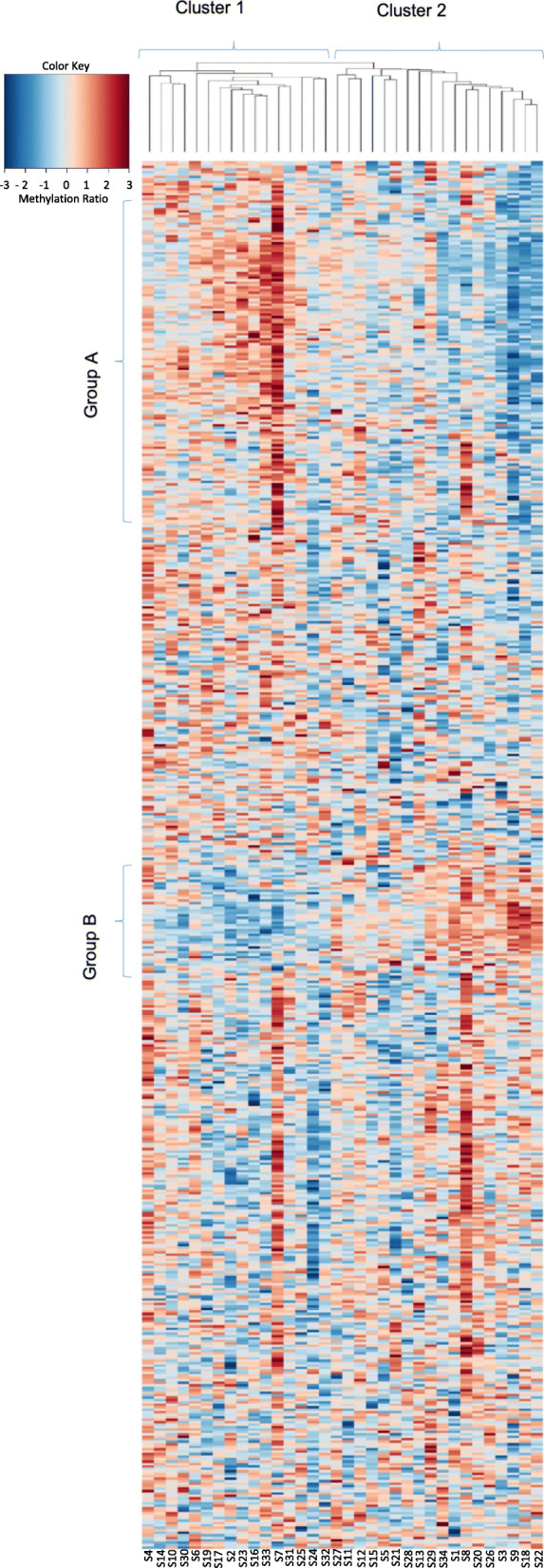
Heatmap showing average methylation ratio for all samples and genes. Scale: Red indicates high methylation and blue indicate low methylation

For each sample, we calculated the mean methylation for each of the 565 capture regions based on individual CpG methylation ratios within each actual region (see the “Methods” section for details). We observed large inter-region variation in the methylation levels of the regions within the 283 tumor suppressor gene promoters analyzed (Fig. 2). Some regions were completely methylated (e.g., regions within the promoters of *AIP*, *PRDM2*, *ATR*, *DICER1*, *SFPQ*), while others in general were non-methylated in most individuals (e.g., regions within *ARID2*, *TRIM33*, *SETD2*, *IKZF1*, and *ARID1B*; Supplementary Figure 1).

In some regions, there was a large variability between CpGs within the promoter region, indicating that some CpGs may be constitutively methylated, while others (perhaps more crucial for transcriptional regulation) had a lower methylation level and may be more dynamically methylated (Supplementary Figure 2).

Constitutional promoter hyper-methylation has been classified either as secondary due to a rare genetic/SNP variant [16], typically resulting in high methylation levels [31, 32] or primary, in which case, methylation may occur at a low mosaic level (VAF of < 10%) [23]. As for both cases, we may not expect identifying several affected individuals in a small dataset like the present; thus, lack of differential methylation here may not exclude a gene as a potential epigenetic pathogenic factor. Still, to validate the feasibility of our method, we aimed at exploring potential differential methylation between individuals across our data set. To do so, we took three approaches: first, we assessed differential methylation across the dataset in general. Second, we specifically assessed for individual hyper-methylation, assuming this to be the most relevant alteration regarding inactivating tumor suppressors. Third, we specifically assessed those tumor suppressors where previous data have linked promoter methylation to cancer risk.

### Differential methylation

Subsequent to methylation calling, we identified promoter regions differentially methylated across our sample set. Although low levels of methylation (allele methylation frequency of < 5%) have been shown to affect cancer risk [23], in the present sample set we focused on identifying those genes presenting the largest inter individual methylation variation as a proof-of-concept for our methodological approach. We defined methylation variation in a region according to the difference in absolute but also relative methylation level. First, we assessed the difference in absolute methylation as the difference in percentage of alleles methylated between individual (i.e., difference presented as percent points). Second, we assessed the relative difference between individuals, i.e., the ratio between the highest and lowest methylated individual with respect to percentage of methylated alleles.

Based on a Z-score assessment of a methylation matrix consisting of averaged methylation ratios for each of the 565 capture regions across all 34 samples (see the “Methods” section for details), we identified 206 regions (within the promoters of 149 genes) where a minority (one-third or less) of the samples analyzed were significantly differentially methylated as compared to the majority of samples at a ≥ 99% confidence level (i.e., outside the 99% confidence interval; Supplementary Table S3). Assessing the difference between the samples with the highest and the lowest level of methylation within these 206 regions, about half of the regions (*n* = 101) displayed less than 5 percent point difference. However, several of the tumor suppressor regions displayed a large variation in methylation, with 72 regions displaying > 10 percent point difference and 22 regions displaying > 20 percent points difference between the highest and the lowest methylated samples (Table 2). The largest difference was observed for *GAS7*, where the difference between the highest and the lowest methylated sample was 66.6 percent points.

**Table 2:** Differentially methylated genes. Gene regions with > 20 percent points difference in methylation ratio, between least methylated sample to most methylated sample along with fold change differences are listed. Hyper-methylated target region of those genes are shown in bold

Gene name	Gene capture region	Min. methylation ratio	Max. methylation ratio	Difference in methylation ratio	Fold change
*GAS7*	chr17: 10199716 - 10200316	0.2670	0.9332	0.6662	3.4951
*ELAC2*	chr17: 13019069 - 13019845	0.4581	0.8332	0.3751	1.8188
*GSTM1*	chr1: 109686327 - 109687046	0.6438	1.0000	0.3562	1.5533
*THBS1*	chr15: 39579298 - 39579871	0.4671	0.7885	0.3214	1.6881
***CIITA***	**chr16: 10874982 - 10875928**	**0.2511**	**0.5577**	**0.3066**	**2.221**
***RASSF1***	**chr3: 50339388 - 50340021**	**0.1786**	**0.4720**	**0.2934**	**2.6428**
***CHN1***	**chr2: 174846842 - 174848034**	**0.2141**	**0.5074**	**0.2933**	**2.3699**
*MSH2*	chr2: 47401613 - 47402319	0.5897	0.8734	0.2838	1.4811
*PALB2*	chr16: 23642511 - 23643136	0.6333	0.9134	0.2801	1.4423
*RUNX3*	chr1: 24964233 - 24965550	0.3920	0.6479	0.2559	1.6528
*TP63*	chr3: 189789769 - 189790448	0.6612	0.9059	0.2446	1.3701
***PDCD1LG2***	**chr9: 5510022 - 5511326**	**0.3182**	**0.5511**	**0.2330**	**1.7319**
*AIP*	chr11: 67481632 - 67482276	0.6716	0.9002	0.2286	1.3404
*GPC3*	chrX: 133986729 - 133987434	0.5842	0.8036	0.2194	1.3756
*AIP*	chr11: 67482202 - 67482880	0.1035	0.3214	0.2180	3.1053
***GSTP1***	**chr11: 67581895 - 67582976**	**0.2673**	**0.4834**	**0.2162**	**1.8085**
*AIP*	chr11: 67481257 - 67481869	0.7857	1.0000	0.2143	1.2728
***XPA***	**chr9: 97698585 - 97699193**	**0.6991**	**0.9130**	**0.2139**	**1.306**
*APC*	chr5: 112736082 - 112736959	0.6361	0.8479	0.2118	1.333
*CTCFL*	chr20: 57524096 - 57527440	0.6554	0.8663	0.2109	1.3218
*CASP8*	chr2: 201259179 - 201260169	0.3698	0.5799	0.2102	1.5681
***ZNF668***	**chr16: 31064314 - 31065859**	**0.4513**	**0.6584**	**0.2070**	**1.4589**
--	--	--	--	--	--
***RABEP1***	**chr17: 5281240 - 5283045**	**0.0415**	**0.1033**	**0.0618**	**2.4902**
***AIP***	**chr11: 67482382 - 67483805**	**0.0517**	**0.1229**	**0.0712**	**2.3783**
***RASSF1***	**chr3: 50338258 - 50339618**	**0.0976**	**0.2178**	**0.1202**	**2.2322**
*FOXO4*	chrX: 71094692 - 71096928	0.1256	0.2592	0.1335	2.0629
*ZRSR2*	chrX: 15789350 - 15791219	0.0559	0.1021	0.0462	1.8252
***RUNX1T1***	**chr8: 92102449 - 92105016**	**0.0558**	**0.1008**	**0.0450**	**1.8068**
***RHOH***	**chr4: 40196452 - 40197679**	**0.1059**	**0.1914**	**0.0855**	**1.8067**

Assessing the relative difference (ratio between the highest and lowest methylated sample), again *GAS7* was the top-ranking promoter, showing a relative difference of 3.5 fold between the highest and the lowest methylated sample. As expected, in addition to *GAS7*, we found a substantial overlap between top-ranking regions based on absolute differences and the top-ranking regions based on relative differences (ratio) in methylation levels (Table 2). Especially the *AIP* gene also had a region that was highly differentially methylated both in terms of percentage difference (> 20%) and fold difference (> 3 fold). The only regions with less than 20 percent point difference but a high fold difference (> 2 fold), were regions in *RABEP1*, *RASSF1*, *AIP*, and *FOXO4* (Table 2, lower section).

### Hyper-methylated tumor suppressors

Regarding tumor suppressor genes, we hypothesized that in case constitutional methylation is associated with a significantly elevated cancer risk, we may expect a minor sub-fraction of healthy individuals to have hyper-methylated promoters. We therefore performed additional sub-analyses restricting 206 genes identified above, to the genes/region with positive Z-scores with > 99% confidence level, i.e., genes/regions that were significantly hyper-methylated in a minority of individuals as compared to the majority of individuals (see the “Methods” section). Among the 206 differentially methylated regions, 115 revealed positive Z-scores. Out of these 115, 25 displayed > 10 percent points difference from the highest to the lowest methylated sample. The corresponding number of regions revealing > 20 percent points difference was 7. These 7 regions were within the promoters of *CIITA*, *RASSF1*, *CHN1*, *PDCD1LG2*, *GSTP1*, *XPA*, and *ZNF668*, with the three former genes revealing a difference of more than 30 percent points (Table 2). Re-assessing these data based on fold difference instead of percent points, we identified three regions (in *AIP*, *RABEP1*, and *RASSF1*) with a lower than 20 percent point absolute difference but a relative ratio > 2. Since another region of *RASSF1* was already identified as having a difference > 20 percent points, this left us with 9 different genes with substantial differences in methylation levels.

Further, we reasoned that if methylation of any of these genes may act as a cancer risk factor, then somatic methylation of the same genes should be present in a fraction of human cancers. We therefore mined the COSMIC data base [33] for reported somatic methylation of the 9 genes. Six of these genes (*CHN1*, *PDCD1LG2*, *XPA*, *ZNF668, RABEP1*, *AIP*) were not reported to be aberrantly somatically methylated in tumors, while one gene (*CIITA*) was reported to be *hypo*-methylated in a very small fraction (0.19-1.53%) of various solid tumors. In contrast, somatic *hyper*-methylation of *RASSF1* was reported in > 4% of endometrial cancers and > 1% of breast cancers. Further, somatic *hyper*-methylation of *GSTP1* was reported in > 7% of prostate cancers and > 1% of breast cancers. Thus, this finding indicates that some genes found hyper-methylated in tumor tissue are also differentially methylated in normal tissue of healthy individuals. Although these data do not provide any conclusive evidence per se, the findings warrant further investigations exploring constitutional methylation as a potential cause of cancer risk.

### Methylation in established cancer risk genes

Among some of the best-characterized cancer risk genes in terms of mutations (*BRCA1*, *TP53*, and *RB1*), we found the mean methylation level to be 0.7% in the known regulatory region of the *BRCA1* promoter, in line with our previous findings [23]. For *TP53*, the mean methylation level was 7.9%, while the corresponding number for *RB1* was 24.9%. For some additional genes where methylation has been found as a cancer risk factor, *MLH1* and *MGMT*, these revealed mean methylation levels of 6.4% and 18.6%, respectively. Among these established cancer risk genes (*BRCA1*, *TP53*, *RB1*, *MLH1*, and *MGMT*), we found no significant differences between the individuals in the present data set.

### Co-methylated tumor suppressors

The cause of differential DNA methylation, and, in particular, tumor suppressor promoter methylation, remains poorly understood. Thus, in an exploratory analysis, we assessed potential covariation between promoter methylation on an individual basis. For this purpose, we performed hierarchical clustering of the samples by applying the Z-scores from average methylation ratio across the 565 capture regions. Doing so, all samples could be classified into two distinct major clusters, each harboring distinguishable sub-clusters (Fig. 2). Interestingly, the two major clusters (1 and 2) were characterized by different promoter methylation in two groups of genes (A and B), where cluster 1 had high methylation in genes in group A and low methylation in genes in group B, while the opposite methylation pattern was seen for samples in cluster 2 (Fig. 2).

We identified genes falling into these two groups (A and B), and analyzed their involvement in functional pathways by KEGG pathway analysis and GO enrichment analysis via Gather. Many of the genes involved in group A were important in development and regulation of cellular processes like Wnt signaling and TGF-beta signaling pathways. In contrast, genes from group B showed involvement in apoptotic pathways and leukocyte differentiation (Supplementary Table S4).

Notably, some individuals were characterized by having a majority of genes either *hyper-* or *hypo*-methylated as compared to the rest of individuals. Applying a 95% confidence interval across samples with respect to the overall methylation level of the regions analyzed, one sample (S24) fell below the lower limit of the CI, while three fell above the upper limit of the CI (Supplementary Figure 3). However, these individuals were distributed across the two main clusters with no preference for one group over the other. Assessing the available general clinical data for these individuals, no notable associations were observed between methylation and factors such as age or BMI (data not shown).

### Validations in external data sets

Although our data are unique since they are generated by targeted massive parallel sequencing analyses, we sought to validate our biological findings by mining available data sets generated by application of methylation arrays.

A technical concern is that methylation could potentially vary between subfractions of leukocytes and differential methylation between individuals could then potentially be a result of individuals having different compositions of leukocyte subfractions in their blood. Assessing the 7 most differentially methylated regions in our data set, in the leukocyte subfractions published in the Bioconductor Experiment Data Package FlowSorted.Blood.450K revealed no major difference in any of the 7 regions (Supplementary Table S5, with figures). In *GSTP1*, 6 out of 19 CpGs revealed lower methylation in CD14+ T cells and/or CD56+ NK cells than other subfractions, but the impact of this on the average levels in total WBC was negligible. Very similar observations were made in another data set of cord blood (R package FlowSorted. CordBlood Norway.450 K in Bioconductor [34]; Supplementary Table S6, with figures). This confirmed potentially varying composition of leukocyte subfractions not to be a likely cause of the observed methylation differences.

Further, we sought to validate the biological differences observed for the 7 most differentially methylated regions in our sample set, by assessing their methylation in a sample of blood DNA from 845 individuals (GSE51032). In this sample set, data was available for *CHN1*, *PDCD1LG2*, *GSTP1*, and *ZNF668*. In addition, we here included the two top-ranking genes with high differential methylation calculated as ratio, but where percent point difference was below 20 (see above; *RABEP1* and *AIP*; Table 2). In general, the methylation levels were called as slightly higher in the GSE51032 set than by our own sequencing. However, the differences between individuals were confirmed for all genes and the difference in percent points between the highest and lowest methylated individual was similar (Supplementary Table S7). The exception was *ZNF668*, where our maximum observation was 66% methylation, while in the GSE51032 set, some individuals were scored as 100% methylated. This difference probably relates to a substantially higher number of individuals analyzed in the validation set increasing the chance of observing outliers.

## Discussion

While to this end constitutional epimutations of tumor suppressors have been linked to cancer risk for a few genes only [23, 27, 31, 35–37], one may postulate that constitutional epimutations affect other tumor suppressors as well. This may have implications to our understanding of cancer risk. A substantial number of cancer-prone families in which no underlying germline mutation have been identified, and it is tempting to postulate that some of these individuals may be at increased cancer risk due to constitutional epimutations in tumor suppressor genes [30]. In addition, germline mutations in several tumor suppressor genes have been associated with other conditions such as skin and limb development deficiencies, Cowden syndrome, and Fanconi anemia [38–40]. Thus, exploring constitutional promoter methylation across tumor suppressor genes may be of importance to other medical conditions as well.

To this end, the vast majority of epigenetic data reported in respect to different health conditions are based on global methylation-array analyses or single gene promoter analyses by methods like MSP or MLPA. While the array-based approaches do provide data for single CpGs, a large number of (potentially important) CpGs are lacking from the arrays, limiting the possibilities to identify methylation pattern across all regions of interest (e.g., as seen for *BRCA1* [23]). As for MSP and MLPA, such methods are fast and cheap but they are sensitive only to a general methylation presence in the CpGs covered by the primers and probes, precluding assessment at a single CpG resolution level.

Here, we established a massive parallel sequencing-based approach, enabling base-pair resolution analyses of methylation status in gene promoters. The method provides several advantages as compared to previous methods. First, as compared to conventional methods like MSP and MLPA, our method allows for detailed single-CpG resolution analyses of multiple promoter regions in concert. Second, our method limits both workload and costs compared to application whole-genome methylation sequencing for promoter methylation analysis. Third, the benefit of determining exact methylation levels, instead of binary assessments, has been confirmed in clinical studies [23], underlining the importance of high sensitivity required to detect low-grade mosaic methylation [30]. Fourth, as compared to available array-based approaches, our NGS-assay allows for methylation assessment of all CpGs in the region of interest, not only those covered by array probes. As mentioned above, this proved to be crucial in analyses of the cancer risk associated with mosaic *BRCA1* methylation [23].

In principle, the sequencing of the DNA-libraries we prepared could be run on any Illumina instrument. As such, the method is flexible and scalable. Here, we used the MiSeq instrument due to the rapid run time. In our set-up, we chose to run 8 samples in one run, yielding an average coverage of 189.6x, corresponding to a mean sensitivity limit of 0.53%. Although indicating a very sensitive method, this is an average value, and some regions reveal lower coverage. If needed, however, coverage could be increased in order to improve the sensitivity of the method [23]. Notably, the reproducibility of the assay may vary between the different covered regions. However, we show that the reproducibility is very good even in regions with low levels of methylation. Importantly, the observed technical variation was consistently negligible compared to the biological variations described. Further, we found that technical variations were lower when assessing all CpGs across a given region than when assessing randomized selections of CpGs as “representative” for a region. This emphasizes the value of applying assays where all CpGs in a given region are covered, instead of relying on scattered, selected CpGs.

While constitutional methylation is considered an early life event affecting different germinal layers, methylation status is also prone to environmental influences and other factors and has been found to change during lifetime [41], causing differential methylation of many genes across different tissues [42]. One potentially important caveat when analyzing WBCs as surrogate markers for constitutional methylation is the fact that different leukocyte fractions may harbor different methylation patterns [43]. While such differences, so far, have been linked to global methylation patterns, it remains unclear whether this may represent a problem with respect to specific tumor suppressor methylation. Notably, differential methylation across WBC subfractions was found not be an issue regarding *BRCA1* promoter methylation [23], and in the present study, it was not found to be an issue in the most differentially methylated promoter regions either.

The methylation level of the genes found to confer cancer risk, so far, is highly variable. Regarding *MLH1*, normal cell methylation affecting ~ 50% of the alleles has been reported in a limited number of probands with familial colorectal cancer (for original references, see [30]). Recently, two families with a high breast and ovarian cancer incidence were found to harbor secondary constitutional *BRCA1* methylation, also with a methylation level of ~ 50% [31]. In contrast, about 4% of females in a Caucasian population was found to carry low-level mosaic constitutional *BRCA1* methylation (4-10% of alleles). Among these low-level methylated individuals, the incidence of high-grade serous ovarian cancer was significantly elevated with an odds ratio between 2 and 3 across two large cohorts [23]. As for the method presented here, this has the sensitivity required for exploring both scenarios.

While the limited number of samples analyzed precludes formal assessments of methylation frequency and/or potential correlations to health outcome, importantly, our findings confirm differential constitutional promoter methylation across a panel of tumor suppressor genes in healthy individuals. Interestingly, among those promoter regions found to be hyper-methylated in the normal tissue of some of the analyzed individuals, we found promoters in genes previously reported to be hyper-methylated in tumors (such as *RASSF1* and *GSTP1*). The presence of epigenetic deregulation of a distinct tumor suppressor at the somatic (tumor) level provides no evidence for constitutional methylation of the same gene. However, the examples related to *MLH1* and *BRCA1* suggest that potential relationships may occur for other genes as well. Thus, it is tempting to speculate that, at least some of the genes detected here (e.g., *RASSF1* and *GSTP1*) could be constitutionally methylated and, in such cases, methylated tumor cells may have originated from the constitutionally methylated normal cells [30]. Notably, although not directly comparable to our data, due to a restricted selection of CpGs covered, mining of a large external data set revealed similar interindividual differences largely confirming our findings.

Interestingly the methylation patterns revealed across our gene panel indicated that the individuals analyzed could be classified into two different methylation clusters. These findings should be interpreted with caution due to the limited number of individuals analyzed. However, the fact that the clusters were separated by differential methylation across important biological pathways involving Wnt- and TGF-beta signaling pathways as well as genes involved in apoptotic pathways and leukocyte differentiation indicate potential underlying biological differences to be explored in future studies.

## Conclusions

We provide a relatively fast and affordable strategy for detailed assessments of differential methylation of tumor suppressors. This strategy is attractive in the warranted search for additional tumor suppressors that may be cancer risk factors when methylated in normal tissues.

## Methods

### Samples

The samples analyzed in the present study were from 34 individuals, selected from a set of 114 healthy postmenopausal women previously described [44]. Subsequent to providing informed consent, each individual donated anonymized blood samples in accordance with Norwegian regulations. All women were recruited during routine mammographic screening at Haukeland University Hospital, Bergen, Norway. Individuals with diabetes or other types of endocrine diseases as well as individuals using hormone replacement therapy were excluded. All samples were drawn > 2 years after the last menstrual period. Within the selection of 34 individuals analyzed in the present study, the mean age was 64 years (range 56-71 years) and the mean BMI was 24.8 (range 19.4-39.6) at the time of sample collection.

### DNA isolation

Genomic DNA was extracted from EDTA-whole blood, using QIAamp DNA Mini kit (Qiagen). The procedure was performed according to the manufacturer’s instructions with the exception that 400 μl of whole blood was used as input.

### Selection of tumor suppressor promoter regions

Regions of interest were defined as 356 regions from the promoters of 283 tumor suppressor genes. The selection of genes was based on the cancer gene panel previously described as “CGPv2/3” [45, 46], Roche’s “Comprehensive Cancer Design” as well as a manual literature review, in order to cover all well-established tumor suppressor genes, independent of cancer type. As such, the selection was independent of previous knowledge about methylation status. For each transcription start site (TSS), we designed probes covering a region spanning from −1500 to +500 relative to TSS. Positions of TSS were determined by NCBI and Ensembl-curated transcripts, literature search, and use of the FANTOM5 RNA expression resource (fantom.gsc.riken.jp/5/). Probes for hybridization to the included regions were manufactured by Roche and designed to bind the target DNA of all possible methylation configurations (fully methylated, partially methylated, and completely unmethylated). Importantly, both strands were targeted, in order to enable correction for potential overlap between CpGs and SNPs. By probe design, the 356 target regions were split into 565 capture regions. Full lists of included tumor suppressor genes and target regions are given in Supplementary Table S1.

### Library preparation and methylation sequencing

Processing of the sample libraries was performed using the solution-based bead capture method for enrichment of bisulfite-converted DNA, SeqCap Epi Enrichment System (Roche) according to the user guide (version 1.2).

For each sample, 1 μg DNA isolated from blood was mixed with bisulfite-conversion control (Lambda DNA, negative for methylation). DNA was fragmented to the range of 180-220 bp using Covaris M220 followed by end repair, A-tailing, ligation of index/adapters, and dual size selection. Using the Zymo Research EZ DNA Methylation-Lightning kit, the DNA was bisulfite-converted according to manufactures protocol, and the resulting sample was amplified prior to nanodrop quantification. Based on these measurements, 1 μg bisulfite-converted DNA was put into the hybridization with custom-made probes for 68 h prior to capture by streptavidin-coated beads, extensive washing, and a final library amplification step.

The protocol was combined with the use of a custom-made probe design enabling analysis of only regions of interest (consisting of 356 promoter regions from 283 tumor suppressor genes, described above and in Supplementary Table S1). In addition, the probe set included probes targeting (Lambda DNA for conversion control). The targeted regions were enriched by a bead capturing method that captures both strands of DNA. Purified libraries were pooled, spiked with 10% PhiX, and sequenced on an Illumina MiSeq sequencer, using v2 chemistry and 2 × 100 (200 cycles) paired-end reads. RTA v1.18.54 and MCS v2.5.0.5 software was used to generate data. Eight samples were multiplexed per run, and resulting data were de-multiplexed based on sample-specific indexes attached to the sequencing adaptors. De-multiplexing was run automatically by the MiSeq Reporter software before further processing.

### Methylation calling

**Fig. 3:**
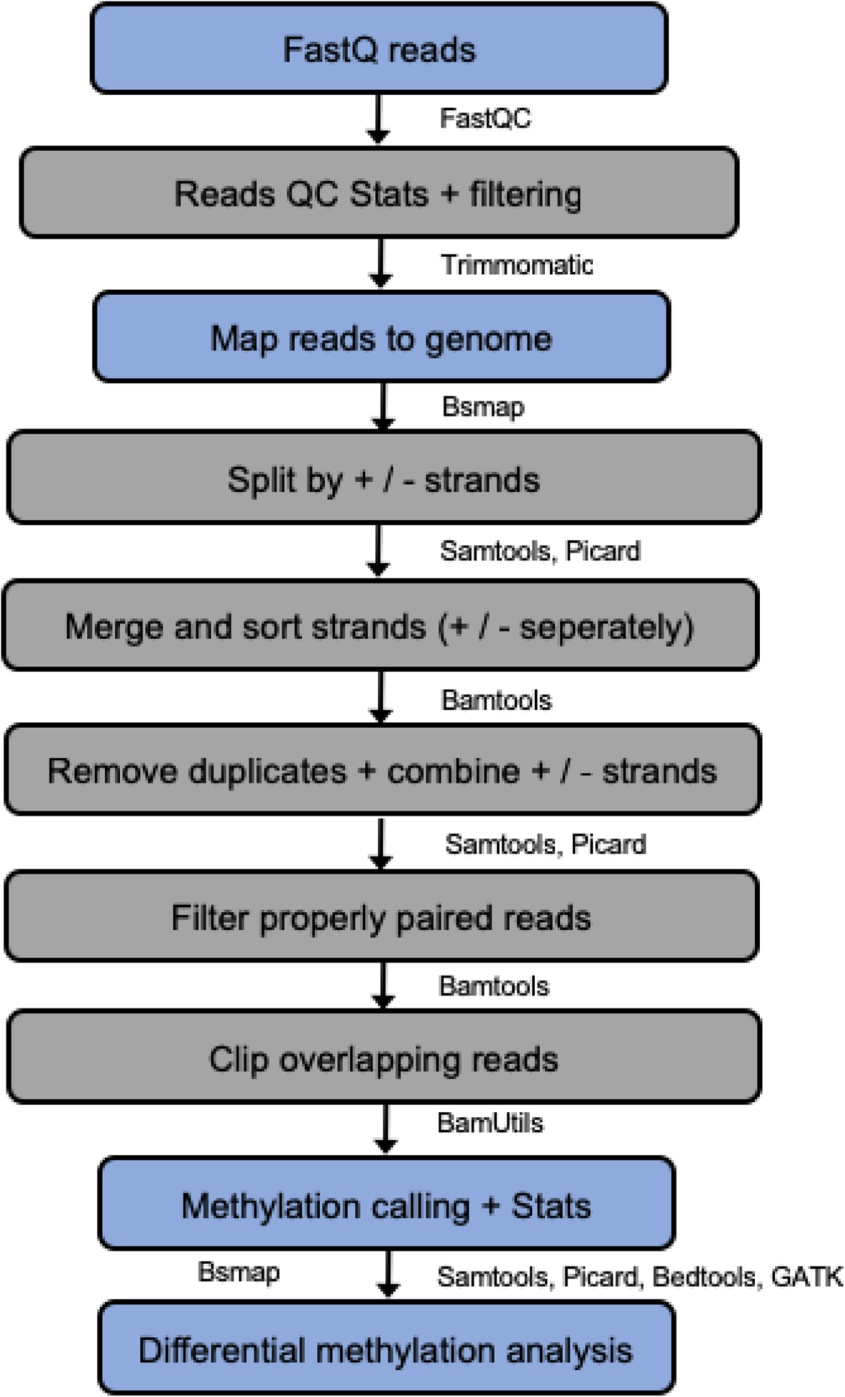
Workflow of the methylation analysis. Flow chart of the steps taken within the informatics analysis pipeline from raw FastQ files to processed data used for biological interpretations. Main steps are indicated by blue background; smaller steps are indicated by gray background (figure adapted from original design by Roche)

Raw sequencing data was analyzed using an in-house workflow designed in collaboration with Roche, comprised of publicly available tools, implemented using shell script (Fig. 3; for a detailed description see Supplementary information). In brief, the first analytic steps involved quality checking of fastq files by FASTQC. Paired-end reads were filtered based on quality and clipped using Trimmomatic [47]. Trimmed sequences were aligned to the human genome (GRCh38) from NCBI as well as Enterobacteria phage lambda (NC_001416.1) complete genome, added for bisulfite conversion efficiency control using the bisulfite mapping algorithm BSMAP [48]. The aligned read statistics and format conversions were carried out using SAMtools [49]. After bisulfite conversion, the DNA strands are no longer complementary. To achieve methylation information from both strands, aligned reads were split into the top and bottom strand [50]. Subsequently, the sequences were sorted, and duplicates were removed and merged back using Picard tools. In the next step, the analysis was further restricted to those read pairs where both mates in the pair could be mapped in the correct orientation and at given distance consistent with the library insert size (properly paired reads) using BamTools [51]. To avoid bias, overlapping reads were clipped using BamUtils. Various statistics for reads, alignment, and coverage were calculated using SamTools.

For each sample, methylation analysis was carried out using methratio.py package in BSMAP by calculating methylation percentage. An additional step involves SNP calling for the targeted regions with BisSNP [52] from aligned reads.

DNA conversion rate was calculated based on all original Cs in the Lambda DNA sequence. For all Cs in the untreated sequence the following formula was used on sequencing data post bisulfite treatment:

$$ \mathrm{Conversion}\ \left(\%\right)=T/\left(C+T\right)\times 100 $$

### Assay reproducibility

To assess reproducibility of the assay, we performed 2 independent experiments with 6 parallels of a standard sample in each experiment. The standard sample consisted of pooled DNA of equal amounts from WBC of 5 healthy donors. Reproducibility was assessed across 12 regions, selected based on three separate criteria: First, we selected 4 regions found to have high biological variance in our original sample set of 34 healthy women (*GAS7*, *ELAC2*, *AIP*, *ZRSR2*). Further, we selected 6 regions in genes known to be high penetrance genes when either mutated or hypermethylated (*BRCA1*, *TP53* (2 regions), *RB1*, *MLH1*, *MGMT*). Finally, we selected 2 regions at random (*PRDM2*, *TMEFF2*). Based on the 12 replicate analyses, we calculated mean methylation, standard deviation and coefficient of variation for all the regions (Supplementary Table S2a). Further, within the 2 randomly selected genes (*PRDM2*, *TMEFF2)*, we performed a randomized selection of 5 CpGs per region, using the mean methylation in these 5 as “representative” for the region. Then, we calculated mean methylation, standard deviation and coefficient of variation across the 12 replicate analyses of these 5 CpGs. This randomization was repeated 5 times, yielding a general overview of the variability when applying limited numbers of CpGs as “representative” for a region (Supplementary Table S2b).

### Differential methylation assessment

Among all CpGs in the 565 capture regions as well as 250 flanking bps at each end, the analysis was restricted only to include CpGs with minimum of 10 reads in coverage in all of the 34 samples. For each sample, we then calculated the mean methylation per region, based on individual CpG methylation ratios within the region. Based on these data, we generated a methylation matrix for all the common regions across all the samples (*n* = 34 in the present study), and calculated Z-scores for that matrix. Then we assessed the Z-scores and identified all the regions where a minority of individuals were differentially methylated as compared to the majority. Differential methylation was here defined as Z-scores that were outside of the 99% confidence interval. We used an arbitrary definition of minority, set to one-third, or less, of the total number of samples, i.e., minimum 1 individual and maximum 12 individuals (this definition may need adjustment according to the size of subsequent studies). Regions that had confidence level more than 99% were then categorized into negatively and positively methylated regions based on the Z-score value and whether the minority of individuals had higher or lower methylation levels than the majority.

To find the differentially methylated regions, we calculated the mean methylation for these regions across CpGs within individual samples and measured the difference in methylation between individuals with the lowest and highest methylation mean. Although relatively small differences in methylation levels have been shown to modulate cancer risk [23], we here sought to identify the regions with larger differences, applying arbitrary thresholds of 5, 10, and 20 percent point difference in methylation. Further, we performed additional analyses assessing ratios (fold difference) between individuals, taking into account that biological important differences may have high ratios, not necessarily reaching a certain threshold set by percent point difference (e.g., a difference between 1% and 10% may be important, even if the percent point difference is only 9).

### Hierarchical clustering

We created a matrix of methylation ratios for all genes across patients. We then calculated a variance for each gene across patients to identify differential methylation. Heatmap was produced with heatplot function from made 4 package [53], with mean linkage cluster analysis and a correlation metric distance. For the purpose of clustering, missing values for regions in individual patients were filled in using the impute R package [54, 55]. (Impute-knn function from impute R package, finds k-nearest neighbors using a Euclidean metric and uses their mean to substitute the missing value). Missing values affected one region of *GSTM1* in 16 samples, another region of *GSTM1* in 7 samples, and a region of *AIP* in 3 samples.

### Pathway analysis

We identified groups of genes from cluster analysis and explored their functional roles by pathway analyses with GATHER. GATHER is an online platform that predicts functional molecular patterns and biological context by incorporation of several biological databases [56]. In GATHER, we analyzed KEGG pathways and gene ontology enrichment analyses [57].

### External data sets

We performed data mining and extracted detailed methylation status for all available CpGs for a given region (defined by our NGS-panel) from the Bioconductor Experiment Data Package FlowSorted.Blood.450K (https://bioconductor.org/packages/release/data/experiment). This data set was generated by methylation array analyses across 6 independent samples from adult individuals and contains information on 10 different categories of leukocytes. The categories include the major groups of granulocytes and lymphocytes.

We obtained similar data for umbilical cord blood from newborns [34]. These data were available as the R package FlowSorted.CordBloodNorway.450K in Bioconductor. This data set was also based on methylation array and holds information about 7 categories of leukocytes, including the major groups of granulocytes and lymphocytes, across 11 independent cord blood samples from newborns.

For validation of methylation differences in blood DNA from healthy individuals, we mined data from GSE51032, available through Gene Expression Omnibus (GEO). This data set was generated by methylation array and consists of 845 samples from the EPIC-Italy cohort (out of which 188 were males and 657 were females).

## Supplementary information

**Additional file 1: Supplementary Figure 1.** Fraction of methylated alleles in promoter region of selected tumour suppressor genes. (A) Regions with high methylation levels across samples from all 34 healthy individuals. (B) Regions with low methylation levels across the same samples. Note the different scale on the Y-axis for panel A and B. Data for AIP were lacking for samples 32, 33, 34 due to low coverage (see details in Materials and methods). **Supplementary Figure 2.** Plot examplifying consistent high and low methylated CpGs in the same promoter, across patients. Fraction of methylated alleles across CpGs in the promoter region of *RB1* in the two samples S7 and S24 are displayed. These two samples were selected because they were the one with highest and lowest overall methylation across the 283 investigated tumour suppressor genes, respectively (ref. Supplementary figure 3), and as such should represent the extremes. Still within the *RB1* promoter, they reveal a very similar pattern of some CpGs being highly methylated, while others are hardly methylated at all. **Supplementary Figure 3.** Distribution of overall average methylation across 283 tumour suppressor gene promoters in 34 healthy individuals. (A) Bars indicate the average fraction of methylated alleles for all CpGs covered per patient. Dotted red lines indicate the upper and lower border of the 95% confidence interval for the average values per patient (CI for individual observations). Sample S24 falls below the lower border of the CI, indicating general *hypo*-methylation. Samples S4, S8 and S7 fall above the upper border of the CI, indicating general *hyper*-methylation. (B) Q-Q plot based on the same data as displayed in (A). S24 is encircled in green, while S4, S8 and S7 are encircled in red.

**Additional file 2: Supplementary information – workflow**

**Additional file 3: Supplementary Table S1.** Pan-cancer panel of 283 tumor suppressor genes for which promoters are included in methylation analyses. The panel was generated based on CGPv2/3-panels [1], Roche’s Comprehensive Cancer Design along with manual literature search.

**Additional file 4: Supplementary Table S2a.** Reproducibility test. **Supplementary Table S2b.** Reproducibility test restricted to randomised CpGs.

**Additional file 5: Supplementary Table S3.** Genes with >99 confidence level difference in methylation ratio between a minority (one third or less) of samples versus the majority.

**Additional file 6: Supplementary Table S4.** groupAB_GE

**Additional file 7: Supplementary Table S5.** WBC fractions

**Additional file 8: Supplementary Table S6.** Coord blood

**Additional file 9: Supplementary Table S7.** EPIC

## Data Availability

All data generated or analyzed during this study are included in this published article (and its supplementary information files). Unprocessed raw files are available from the corresponding author on reasonable request.

## Abbreviations

BMI: Body mass index
CpG: Cytosine-phosphate-guanine
HGSOC: High grade serous ovarian cancer
MSP: Methylation-specific polymerase chain reaction
MLPA: Multiplex ligation-dependent probe amplification
NGS: Next-generation sequencing
SNP: Single nucleotide polymorphism
WBC: White blood cells
