# Supplementary material for: Assessment of tumor suppressor promoter methylation in healthy individuals
Source: Clin Epigenetics. 2020 Aug 28;12:131. doi: 10.1186/s13148-020-00920-7 (PMC7455917; doi:10.1186/s13148-020-00920-7)
Supplement: Supplementary file 1 — Additional file 1: Supplementary Figure 1. Fraction of methylated alleles in promoter region of selected tumour suppressor genes. (A) Regions with high methylation levels across samples from all 34 healthy individuals. (B) Regions with low methylation levels across the same samples. Note the different scale on the Y-axis for panel A and B. Data for AIP were lacking for samples 32, 33, 34 due to low coverage (see details in Materials and methods). Supplementary Figure 2. Plot examplifying consistent high and low methylated CpGs in the same promoter, across patients. Fraction of methylated alleles across CpGs in the promoter region of RB1 in the two samples S7 and S24 are displayed. These two samples were selected because they were the one with highest and lowest overall methylation across the 283 investigated tumour suppressor genes, respectively (ref. Supplementary figure 3), and as such should represent the extremes. Still within the RB1 promoter, they reveal a very similar pattern of some CpGs being highly methylated, while others are hardly methylated at all. Supplementary Figure 3. Distribution of overall average methylation across 283 tumour suppressor gene promoters in 34 healthy individuals. (A) Bars indicate the average fraction of methylated alleles for all CpGs covered per patient. Dotted red lines indicate the upper and lower border of the 95% confidence interval for the average values per patient (CI for individual observations). Sample S24 falls below the lower border of the CI, indicating general hypo-methylation. Samples S4, S8 and S7 fall above the upper border of the CI, indicating general hyper-methylation. (B) Q-Q plot based on the same data as displayed in (A). S24 is encircled in green, while S4, S8 and S7 are encircled in red. [file 13148_2020_920_MOESM1_ESM.docx]

A


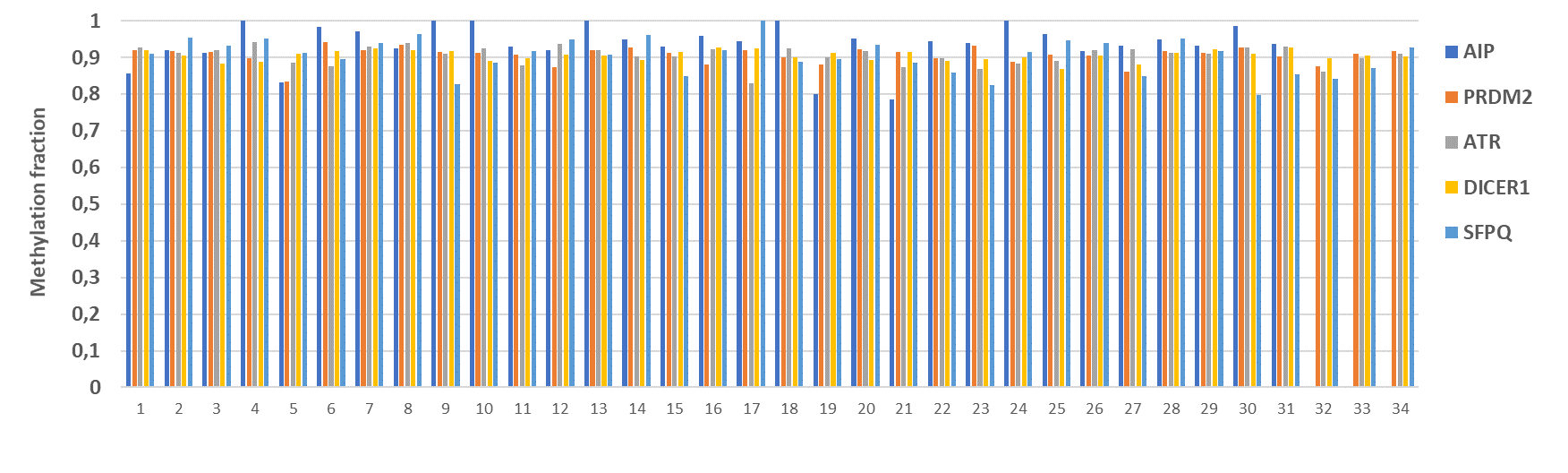


B


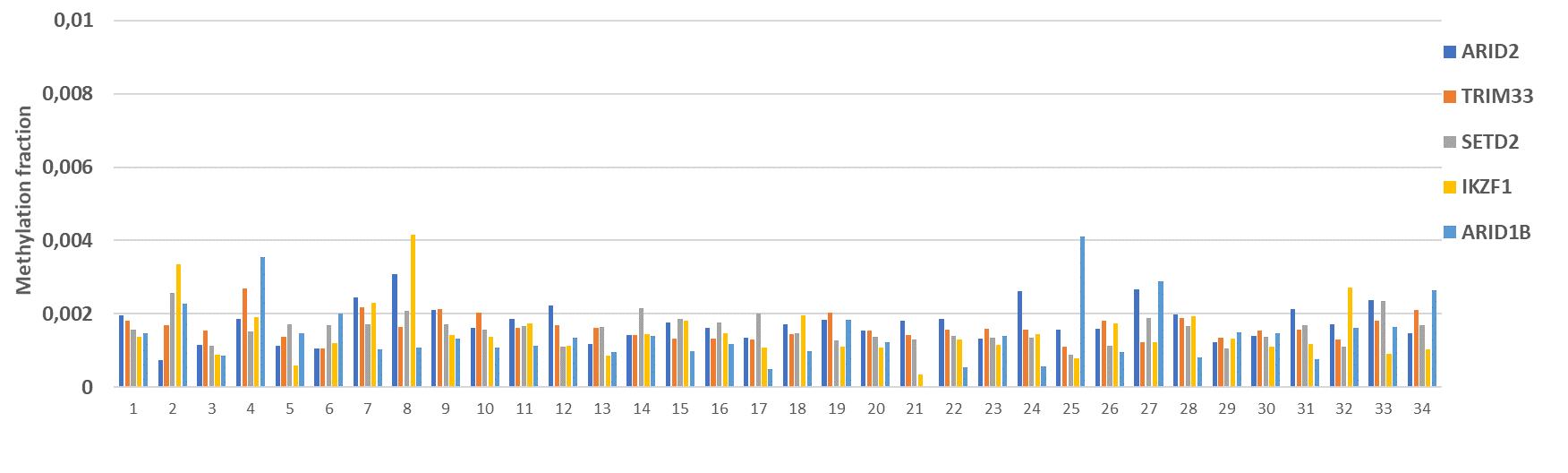


**Supplementary figure 1.** Fraction of methylated alleles in promoter region of selected tumour suppressor genes. (A) Regions with high methylation levels across samples from all 34 healthy individuals. (B) Regions with low methylation levels across the same samples. Note the different scale on the Y-axis for panel A and B. Data for AIP were lacking for samples 32, 33, 34 due to low coverage (see details in Materials and methods).


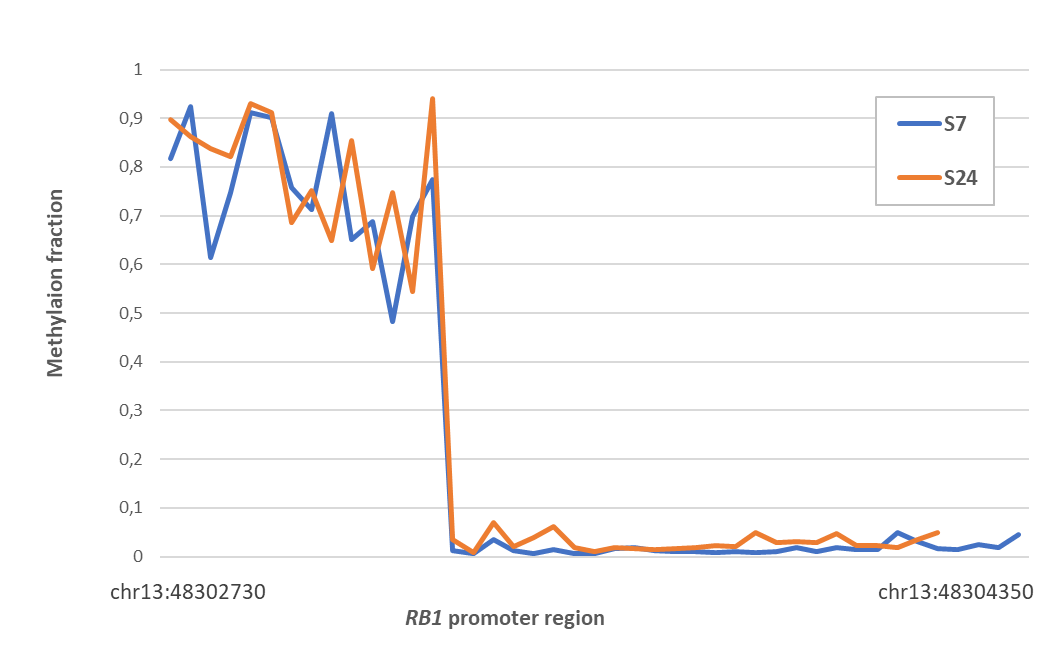


**Supplementary figure 2.** Plot examplifying consistent high and low methylated CpGs in the same promoter, across patients. Fraction of methylated alleles across CpGs in the promoter region of *RB1* in the two samples S7 and S24 are displayed. These two samples were selected because they were the one with highest and lowest overall methylation across the 283 investigated tumour suppressor genes, respectively (ref. Supplementary figure 3), and as such should represent the extremes. Still within the *RB1* promoter, they reveal a very similar pattern of some CpGs being highly methylated, while others are hardly methylated at all.

A


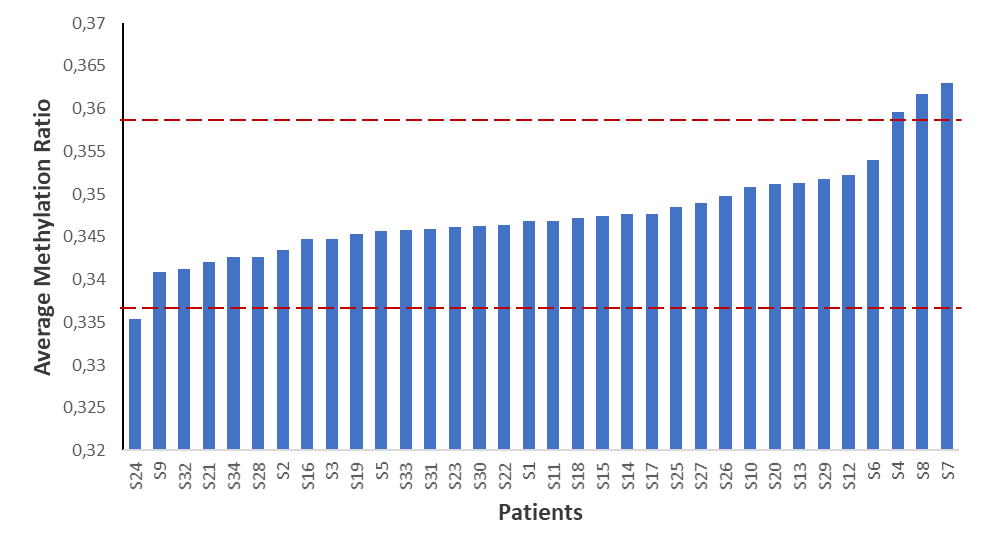


B


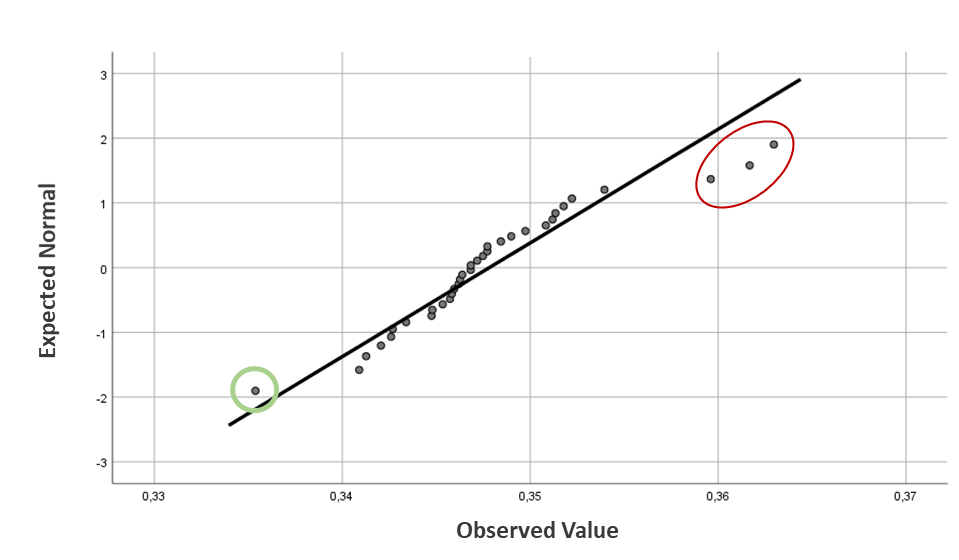


**Supplementary figure 3.** Distribution of overall average methylation across 283 tumour suppressor gene promoters in 34 healthy individuals. (A) Bars indicate the average fraction of methylated alleles for all CpGs covered per patient. Dotted red lines indicate the upper and lower border of the 95% confidence interval for the average values per patient (CI for individual observations). Sample S24 falls below the lower border of the CI, indicating general hypomethylation. Samples S4, S8 and S7 fall above the upper border of the CI, indicating general hypermethylation. (B) Q-Q plot based on the same data as displayed in (A). S24 is encircled in green, while S4, S8 and S7 are encircled in red.
