# Supplementary material for: Assessment of tumor suppressor promoter methylation in healthy individuals
Source: Clin Epigenetics. 2020 Aug 28;12:131. doi: 10.1186/s13148-020-00920-7 (PMC7455917; doi:10.1186/s13148-020-00920-7)
Supplement: Supplementary file 2 — Additional file 2: Supplementary information – workflow [file 13148_2020_920_MOESM2_ESM.docx]

**Supplementary information – workflow.**

In order to analyze captured raw sequencing reads we designed an in-house workflow in collaboration with Roche, based on their description provided in [Tech Note_How to Evaluate SeqCap Epi Target Enrichment Data_v1.1.pdf](https://rochedia.showpad.com/share/56LRpYiv7Q9C1rnzB2NKq/0) (see also Figure 1 for detailed description). The workflow is implemented in a shell script comprised of tools available in public domain. The workflow starts with quality control of sequenced data using FASTQC. Next step involved trimming of reads based on their quality as well as Illumina adapters using Trimmomatic [1]. We then align trimmed sequences to human genome (GRCh38) from NCBI as well as Enterobacteria phage lambda (NC_001416.1) complete genome with help of efficient bisulfite mapping algorithm BSMAP [2]. Lamda genome was used in order to measure quality of bisulfite conversion efficiency. Alignment statistics and format conversion for further downstream process are carried out using SAMtools [3]. Methylation information was obtained from both strands (top and bottom strand) of aligned reads as DNA strands are no longer complementary after bisulfite conversion [4]. All of the strands were sorted before removing duplicates and merged back using Picard tools. To ensure more stringency in the analysis, in the next step reads were restricted to those with proper paired end reads using BamTools [5]. This was achieved by considering only reads pairs that were identified as pairs and at the same time mapped at correct distance and mapped in correct orientation with properly paired library insert sizes. In the next step, overlapping reads were clipped to avoid bias using BamUtils. Different metrics for filtered reads, and coverage were assessed using SamTools. For each sample, methylation analysis involved calculation of methylation percentage using methratio.py package in BSMAP. We also included a step for SNP calling in targeted regions using BisSNP [6].

*Execution*

The workflow was designed to assess differentially methylated regions from targeted bisulfite sequencing of our panel of regions Sequencing data (fastq) was used as input. The script (separate Supplementary file) was run by the command:

- sh SeqCap_Epi_nor_univ_bergen_final.sh output_folder_name/ tumor_file_folder/ normal_file_folder/

The resulting output folder holds all output files, including alignment statistics etc. By default, when run as pairwise comparisons, the methylation result text file includes all differentially methylated regions with more than 25 percent points methylation difference. This is adjustable according to needs / aims of the study.

1. Bolger AM, Lohse M, Usadel B. Trimmomatic: a flexible trimmer for Illumina sequence data. Bioinformatics. 2014;30(15):2114-20. Epub 2014/04/04. doi: 10.1093/bioinformatics/btu170. PubMed PMID: 24695404; PubMed Central PMCID: PMCPMC4103590.

2. Xi Y, Li W. BSMAP: whole genome bisulfite sequence MAPping program. BMC Bioinformatics. 2009;10:232. Epub 2009/07/29. doi: 10.1186/1471-2105-10-232. PubMed PMID: 19635165; PubMed Central PMCID: PMCPMC2724425.

3. Li H. A statistical framework for SNP calling, mutation discovery, association mapping and population genetical parameter estimation from sequencing data. Bioinformatics. 2011;27(21):2987-93. Epub 2011/09/10. doi: 10.1093/bioinformatics/btr509. PubMed PMID: 21903627; PubMed Central PMCID: PMCPMC3198575.

4. Law JA, Jacobsen SE. Establishing, maintaining and modifying DNA methylation patterns in plants and animals. Nature reviews Genetics. 2010;11(3):204-20. doi: 10.1038/nrg2719. PubMed PMID: 20142834; PubMed Central PMCID: PMCPMC3034103.

5. Barnett DW, Garrison EK, Quinlan AR, Stromberg MP, Marth GT. BamTools: a C++ API and toolkit for analyzing and managing BAM files. Bioinformatics. 2011;27(12):1691-2. Epub 2011/04/16. doi: 10.1093/bioinformatics/btr174. PubMed PMID: 21493652; PubMed Central PMCID: PMCPMC3106182.

6. Liu Y, Siegmund KD, Laird PW, Berman BP. Bis-SNP: Combined DNA methylation and SNP calling for Bisulfite-seq data. Genome biology. 2012;13(7):1-14. doi: 10.1186/gb-2012-13-7-r61.
