# Supplementary material for: Assessment of tumor suppressor promoter methylation in healthy individuals
Source: Clin Epigenetics. 2020 Aug 28;12:131. doi: 10.1186/s13148-020-00920-7 (PMC7455917; doi:10.1186/s13148-020-00920-7)
Supplement: Supplementary file 3 — Additional file 3: Supplementary Table S1. Pan-cancer panel of 283 tumor suppressor genes for which promoters are included in methylation analyses. The panel was generated based on CGPv2/3-panels [1], Roche’s Comprehensive Cancer Design along with manual literature search. [file 13148_2020_920_MOESM3_ESM.docx]

**Supplementary Table S1**. Pan-cancer panel of 283 tumor suppressor genes for which promoters are included in methylation analyses. The panel was generated based on CGPv2/3-panels[1], Roche’s Comprehensive Cancer Design along with manual literature search.

| **Gene Name** | **Chromosome** | **Start position** | **End position** |
| --- | --- | --- | --- |
|  |  |  |  |
| AIP | chr11 | 67481257 | 67481869 |
| AIP | chr11 | 67481632 | 67482276 |
| AIP | chr11 | 67482202 | 67482880 |
| AIP | chr11 | 67482382 | 67483805 |
| ALDH2 | chr12 | 111765396 | 111766262 |
| ALDH2 | chr12 | 111766026 | 111767662 |
| AMER1 | chrX | 64204967 | 64207518 |
| APC | chr5 | 112705736 | 112708295 |
| APC | chr5 | 112736082 | 112736959 |
| APC | chr5 | 112736659 | 112738634 |
| AR | chrX | 67542261 | 67544804 |
| AR | chrX | 67567065 | 67569614 |
| ARHGAP26 | chr5 | 142768948 | 142771512 |
| ARHGEF12 | chr11 | 120335132 | 120337693 |
| ARHGEF12 | chr11 | 120383513 | 120385133 |
| ARHGEF12 | chr11 | 120384638 | 120386069 |
| ARID1A | chr1 | 26694264 | 26696809 |
| ARID1B | chr6 | 156776159 | 156778458 |
| ARID1B | chr6 | 156778004 | 156778717 |
| ARID2 | chr12 | 45728058 | 45729857 |
| ARID2 | chr12 | 45729404 | 45730622 |
| ARID4A | chr14 | 58296723 | 58299283 |
| ASXL1 | chr20 | 32356589 | 32359117 |
| ATM | chr11 | 108221059 | 108223605 |
| ATR | chr3 | 142578047 | 142580131 |
| ATR | chr3 | 142579817 | 142580609 |
| ATRX | chrX | 77785489 | 77787170 |
| ATRX | chrX | 77786686 | 77787576 |
| ATRX | chrX | 77787321 | 77788061 |
| AXIN2 | chr17 | 65560843 | 65562936 |
| AXIN2 | chr17 | 65562733 | 65563403 |
| BAP1 | chr3 | 52409336 | 52411884 |
| BARD1 | chr2 | 214808939 | 214811496 |
| BCL7A | chr12 | 122020383 | 122022184 |
| BCL7A | chr12 | 122021689 | 122022739 |
| BLID | chr11 | 122115435 | 122117985 |
| BLM | chr15 | 90715550 | 90716525 |
| BLM | chr15 | 90716100 | 90718096 |
| BMP2 | chr20 | 6766326 | 6768883 |
| BMP3 | chr4 | 81029188 | 81029875 |
| BMP3 | chr4 | 81029639 | 81030688 |
| BMP3 | chr4 | 81030219 | 81031752 |
| BMP4 | chr14 | 53953780 | 53956333 |
| BMP4 | chr14 | 53956060 | 53958618 |
| BMP7 | chr20 | 57265875 | 57268435 |
| BMPR1A | chr10 | 86754872 | 86757419 |
| BRCA1 | chr17 | 43123372 | 43125000 |
| BRCA1 | chr17 | 43124700 | 43127003 |
| BRCA1 | chr17 | 43126542 | 43127258 |
| BRCA2 | chr13 | 32313702 | 32314835 |
| BRCA2 | chr13 | 32314559 | 32316256 |
| BRIP1 | chr17 | 61862781 | 61865132 |
| BTG1 | chr12 | 92145118 | 92147131 |
| BTG1 | chr12 | 92146634 | 92147458 |
| BTG1 | chr12 | 92146959 | 92147652 |
| BUB1B | chr15 | 40159231 | 40160455 |
| BUB1B | chr15 | 40160045 | 40161786 |
| CARS | chr11 | 3056672 | 3058337 |
| CARS | chr11 | 3057962 | 3058553 |
| CARS | chr11 | 3058342 | 3059232 |
| CASC5 | chr15 | 40592469 | 40595032 |
| CASP8 | chr2 | 201231670 | 201233251 |
| CASP8 | chr2 | 201232785 | 201234218 |
| CASP8 | chr2 | 201256253 | 201257147 |
| CASP8 | chr2 | 201256864 | 201257468 |
| CASP8 | chr2 | 201257228 | 201258816 |
| CASP8 | chr2 | 201258724 | 201259427 |
| CASP8 | chr2 | 201259179 | 201260169 |
| CASP8 | chr2 | 201259733 | 201261281 |
| CCDC6 | chr10 | 59905876 | 59908436 |
| CCNB1IP1 | chr14 | 20332533 | 20334430 |
| CCNB1IP1 | chr14 | 20334095 | 20335097 |
| CD2 | chr1 | 116752965 | 116755246 |
| CDC73 | chr1 | 193120186 | 193122737 |
| CDH1 | chr16 | 68735675 | 68736496 |
| CDH1 | chr16 | 68736420 | 68737091 |
| CDH1 | chr16 | 68736695 | 68738068 |
| CDH11 | chr16 | 65121246 | 65123801 |
| CDH13 | chr16 | 82625015 | 82627558 |
| CDK12 | chr17 | 39459732 | 39460822 |
| CDK12 | chr17 | 39460362 | 39461006 |
| CDK12 | chr17 | 39460837 | 39462288 |
| CDK2AP2 | chr11 | 67507407 | 67509678 |
| CDK2AP2 | chr11 | 67509392 | 67510498 |
| CDKN1A | chr6 | 36674681 | 36675697 |
| CDKN1A | chr6 | 36675451 | 36676113 |
| CDKN1A | chr6 | 36675761 | 36677228 |
| CDKN1A | chr6 | 36676906 | 36679480 |
| CDKN1B | chr12 | 12715490 | 12718045 |
| CDKN1C | chr11 | 2884986 | 2887543 |
| CDKN2A | chr9 | 21974060 | 21976361 |
| CDKN2A | chr9 | 21993713 | 21996257 |
| CDKN2B | chr9 | 22008542 | 22011097 |
| CDKN2C | chr1 | 50966917 | 50970751 |
| CDKN2D | chr19 | 10568175 | 10570125 |
| CDKN2D | chr19 | 10569864 | 10570540 |
| CDX2 | chr13 | 27968588 | 27971148 |
| CEBPA | chr19 | 33301785 | 33304351 |
| CHD5 | chr1 | 6179359 | 6181920 |
| CHD6 | chr20 | 41617717 | 41620276 |
| CHEK1 | chr11 | 125623361 | 125624312 |
| CHEK1 | chr11 | 125623875 | 125624579 |
| CHEK1 | chr11 | 125624255 | 125624936 |
| CHEK1 | chr11 | 125624681 | 125626998 |
| CHEK2 | chr22 | 28741055 | 28743614 |
| CHFR | chr12 | 132886841 | 132889287 |
| CHN1 | chr2 | 174846842 | 174848034 |
| CHN1 | chr2 | 174847546 | 174849392 |
| CHN1 | chr2 | 175004476 | 175007018 |
| CIC | chr19 | 42282884 | 42285452 |
| CIITA | chr16 | 10874982 | 10875928 |
| CIITA | chr16 | 10876007 | 10878457 |
| CLDN3 | chr7 | 73769491 | 73771422 |
| CLDN3 | chr7 | 73771154 | 73772013 |
| CLDN4 | chr7 | 73829087 | 73831656 |
| CLTCL1 | chr22 | 19290939 | 19293498 |
| CNBP | chr3 | 129183187 | 129185101 |
| CNBP | chr3 | 129184602 | 129185353 |
| COX6C | chr8 | 99893234 | 99895453 |
| COX6C | chr8 | 99895019 | 99895783 |
| CREB3L1 | chr11 | 46275857 | 46277219 |
| CREB3L1 | chr11 | 46276972 | 46278418 |
| CREBBP | chr16 | 3879342 | 3881900 |
| CTCFL | chr20 | 57524096 | 57527440 |
| CTNNB1 | chr3 | 41197680 | 41200220 |
| CYLD | chr16 | 50740283 | 50741291 |
| CYLD | chr16 | 50740793 | 50742832 |
| DAPK1 | chr9 | 87495448 | 87496206 |
| DAPK1 | chr9 | 87495950 | 87496952 |
| DAPK1 | chr9 | 87496675 | 87499315 |
| DDB2 | chr11 | 47212674 | 47213507 |
| DDB2 | chr11 | 47213951 | 47214815 |
| DDB2 | chr11 | 47214529 | 47216210 |
| DDIT3 | chr12 | 57519751 | 57521452 |
| DDIT3 | chr12 | 57521227 | 57522300 |
| DDX53 | chrX | 22998180 | 22998865 |
| DDX53 | chrX | 22998633 | 23000742 |
| DICER1 | chr14 | 95132724 | 95134217 |
| DICER1 | chr14 | 95133719 | 95135268 |
| DICER1 | chr14 | 95136031 | 95136667 |
| DICER1 | chr14 | 95136276 | 95137349 |
| DICER1 | chr14 | 95137099 | 95138591 |
| DICER1 | chr14 | 95156649 | 95158942 |
| DICER1 | chr14 | 95158704 | 95159791 |
| DKK1 | chr10 | 52312503 | 52315052 |
| DNMT3A | chr2 | 25251544 | 25254071 |
| DNMT3A | chr2 | 25341140 | 25344370 |
| EBF1 | chr5 | 159099003 | 159099774 |
| EBF1 | chr5 | 159099284 | 159100066 |
| EBF1 | chr5 | 159099574 | 159100172 |
| EBF1 | chr5 | 159099689 | 159101555 |
| EIF4A2 | chr3 | 186781792 | 186784353 |
| ELAC2 | chr17 | 13017290 | 13018788 |
| ELAC2 | chr17 | 13018410 | 13019501 |
| ELAC2 | chr17 | 13019069 | 13019845 |
| EMP3 | chr19 | 48323926 | 48324869 |
| EMP3 | chr19 | 48324401 | 48325017 |
| EMP3 | chr19 | 48324661 | 48326149 |
| EP300 | chr22 | 41090841 | 41093391 |
| EPHA5 | chr4 | 65669718 | 65672268 |
| EPHA6 | chr3 | 96812800 | 96815362 |
| EPHA6 | chr3 | 97437816 | 97440379 |
| EPHB6 | chr7 | 142853233 | 142855789 |
| EPHB6 | chr7 | 142860240 | 142861791 |
| EPHB6 | chr7 | 142861293 | 142863543 |
| ERCC1 | chr19 | 45422793 | 45425172 |
| ERCC1 | chr19 | 45424948 | 45425643 |
| ERCC2 | chr19 | 45369813 | 45371325 |
| ERCC2 | chr19 | 45371053 | 45371791 |
| ERCC2 | chr19 | 45371478 | 45372154 |
| ERCC3 | chr2 | 127293408 | 127294718 |
| ERCC3 | chr2 | 127294647 | 127295832 |
| ERCC4 | chr16 | 13918377 | 13919791 |
| ERCC4 | chr16 | 13919312 | 13920935 |
| ERCC5 | chr13 | 102844102 | 102845076 |
| ERCC5 | chr13 | 102844654 | 102845369 |
| ERCC5 | chr13 | 102844882 | 102845734 |
| ERCC5 | chr13 | 102845327 | 102846619 |
| ERG | chr21 | 38497724 | 38500010 |
| ERG | chr21 | 38499589 | 38500284 |
| ERG | chr21 | 38661006 | 38663557 |
| ESR1 | chr6 | 151688716 | 151691278 |
| ESR1 | chr6 | 151803894 | 151806772 |
| ESR1 | chr6 | 151806314 | 151808440 |
| EXT1 | chr8 | 118111041 | 118113596 |
| EXT2 | chr11 | 44093769 | 44094934 |
| EXT2 | chr11 | 44094664 | 44096978 |
| FAM46C | chr1 | 117604214 | 117606766 |
| FANCA | chr16 | 89815877 | 89817388 |
| FANCA | chr16 | 89817237 | 89818203 |
| FANCA | chr16 | 89817712 | 89818428 |
| FANCB | chrX | 14872282 | 14874819 |
| FANCC | chr9 | 95316483 | 95318620 |
| FANCC | chr9 | 95318648 | 95319499 |
| FANCD2 | chr3 | 10024958 | 10025701 |
| FANCD2 | chr3 | 10025203 | 10027217 |
| FANCE | chr6 | 35450583 | 35453141 |
| FANCF | chr11 | 22625068 | 22627198 |
| FANCF | chr11 | 22626801 | 22627613 |
| FANCG | chr9 | 35079240 | 35080574 |
| FANCG | chr9 | 35080079 | 35080717 |
| FANCG | chr9 | 35080399 | 35081139 |
| FANCG | chr9 | 35081169 | 35081293 |
| FANCI | chr15 | 89242185 | 89244752 |
| FANCL | chr2 | 58240644 | 58243159 |
| FANCM | chr14 | 45134154 | 45136711 |
| FAS | chr10 | 88988750 | 88991317 |
| FAT1 | chr4 | 186723053 | 186725613 |
| FBXO11 | chr2 | 47887940 | 47890324 |
| FBXO11 | chr2 | 47905020 | 47907557 |
| FBXW7 | chr4 | 152352181 | 152354741 |
| FBXW7 | chr4 | 152381741 | 152382977 |
| FBXW7 | chr4 | 152382496 | 152384206 |
| FBXW7 | chr4 | 152534255 | 152537023 |
| FH | chr1 | 241519006 | 241521560 |
| FHIT | chr3 | 61250682 | 61253237 |
| FHL1 | chrX | 136144923 | 136149352 |
| FHL1 | chrX | 136167518 | 136170421 |
| FHL1 | chrX | 136194975 | 136197530 |
| FLCN | chr17 | 17236411 | 17238116 |
| FLCN | chr17 | 17237841 | 17238743 |
| FOXL2 | chr3 | 138946361 | 138948919 |
| FOXO1 | chr13 | 40665821 | 40668369 |
| FOXO3 | chr6 | 108558045 | 108561652 |
| FOXO4 | chrX | 71094368 | 71095010 |
| FOXO4 | chrX | 71094692 | 71096928 |
| FUS | chr16 | 31178329 | 31180898 |
| GAS7 | chr17 | 10025528 | 10028002 |
| GAS7 | chr17 | 10035978 | 10038207 |
| GAS7 | chr17 | 10197776 | 10199919 |
| GAS7 | chr17 | 10199716 | 10200316 |
| GAS7 | chr17 | 9958680 | 9960456 |
| GAS7 | chr17 | 9960239 | 9961233 |
| GATA1 | chrX | 48784803 | 48787354 |
| GATA2 | chr3 | 128487147 | 128490318 |
| GATA2 | chr3 | 128492407 | 128494942 |
| GATA3 | chr10 | 8052925 | 8055161 |
| GATA3 | chr10 | 8054667 | 8055490 |
| GATA4 | chr8 | 11702430 | 11704993 |
| GATA5 | chr20 | 62475199 | 62477145 |
| GATA5 | chr20 | 62476706 | 62477292 |
| GMPS | chr3 | 155868983 | 155869898 |
| GMPS | chr3 | 155869403 | 155871317 |
| GPC3 | chrX | 133984871 | 133986973 |
| GPC3 | chrX | 133986729 | 133987434 |
| GSTM1 | chr1 | 109686097 | 109686745 |
| GSTM1 | chr1 | 109686327 | 109687046 |
| GSTM1 | chr1 | 109686642 | 109687492 |
| GSTM1 | chr1 | 109686997 | 109688572 |
| GSTP1 | chr11 | 67581895 | 67582976 |
| GSTP1 | chr11 | 67582510 | 67583218 |
| GSTP1 | chr11 | 67583140 | 67584374 |
| HAND2 | chr4 | 173529447 | 173532007 |
| HECW1 | chr7 | 43110821 | 43113387 |
| HERPUD1 | chr16 | 56930313 | 56931125 |
| HERPUD1 | chr16 | 56930633 | 56931495 |
| HERPUD1 | chr16 | 56931003 | 56932876 |
| HIC1 | chr17 | 2053320 | 2057100 |
| HNF1A | chr12 | 120976684 | 120977503 |
| HNF1A | chr12 | 120977599 | 120980028 |
| HOXA10 | chr7 | 27173559 | 27176123 |
| HOXA10 | chr7 | 27179484 | 27182033 |
| HOXA11 | chr7 | 27184439 | 27186994 |
| HOXA9 | chr7 | 27164754 | 27167307 |
| ID4 | chr6 | 19835602 | 19838156 |
| IGFBP3 | chr7 | 45920494 | 45923044 |
| IKZF1 | chr7 | 50302328 | 50305230 |
| IKZF1 | chr7 | 50304733 | 50305451 |
| IKZF1 | chr7 | 50306908 | 50309459 |
| IKZF1 | chr7 | 50317278 | 50318546 |
| IKZF1 | chr7 | 50318048 | 50319826 |
| IL21R | chr16 | 27400381 | 27402920 |
| IL21R | chr16 | 27425579 | 27427633 |
| IL21R | chr16 | 27427333 | 27428035 |
| KDM5C | chrX | 53224644 | 53227201 |
| KDM6A | chrX | 44871397 | 44873963 |
| KDSR | chr18 | 63366493 | 63368320 |
| KDSR | chr18 | 63368028 | 63369060 |
| KEAP1 | chr19 | 10502027 | 10503898 |
| KEAP1 | chr19 | 10504033 | 10504794 |
| KEAP1 | chr19 | 10504565 | 10505163 |
| KL | chr13 | 33014657 | 33017215 |
| KLF6 | chr10 | 3784501 | 3787050 |
| KMT2C | chr7 | 152435227 | 152437363 |
| KMT2C | chr7 | 152436898 | 152437784 |
| KMT2D | chr12 | 49054547 | 49057103 |
| LMNA | chr1 | 156080779 | 156083321 |
| LMNA | chr1 | 156113070 | 156115451 |
| LMNA | chr1 | 156124336 | 156125702 |
| LMNA | chr1 | 156125756 | 156126893 |
| LRP5 | chr11 | 68310830 | 68313385 |
| LTBP2 | chr14 | 74611551 | 74613167 |
| LTBP2 | chr14 | 74612956 | 74614085 |
| MAL | chr2 | 95023875 | 95026425 |
| MC1R | chr16 | 89915602 | 89916637 |
| MC1R | chr16 | 89917282 | 89919160 |
| MEN1 | chr11 | 64809783 | 64811416 |
| MEN1 | chr11 | 64811758 | 64812619 |
| MEN1 | chr11 | 64812298 | 64813077 |
| MGMT | chr10 | 129465421 | 129467970 |
| MIR124-1 | chr8 | 9902695 | 9905243 |
| MIR127 | chr14 | 100881198 | 100883763 |
| MIR155 | chr21 | 25572205 | 25574739 |
| MLF1 | chr3 | 158569396 | 158571932 |
| MLH1 | chr3 | 36991573 | 36994561 |
| MLLT11 | chr1 | 151057906 | 151060456 |
| MNX1 | chr7 | 157008657 | 157012437 |
| MRE11A | chr11 | 94493095 | 94495644 |
| MSH2 | chr2 | 47401288 | 47401885 |
| MSH2 | chr2 | 47401613 | 47402319 |
| MSH2 | chr2 | 47402085 | 47403844 |
| MSH6 | chr2 | 47781508 | 47782351 |
| MSH6 | chr2 | 47782060 | 47784730 |
| MTUS2 | chr13 | 29022834 | 29025397 |
| MTUS2 | chr13 | 29426863 | 29429423 |
| MUTYH | chr1 | 45339226 | 45341936 |
| MUTYH | chr1 | 45341574 | 45342247 |
| NBN | chr8 | 89983892 | 89985677 |
| NBN | chr8 | 89985399 | 89986457 |
| NCKIPSD | chr3 | 48685154 | 48687192 |
| NCKIPSD | chr3 | 48686864 | 48687722 |
| NDRG1 | chr8 | 133296524 | 133299086 |
| NF1 | chr17 | 31093152 | 31093829 |
| NF1 | chr17 | 31093597 | 31095703 |
| NF2 | chr22 | 29602075 | 29602991 |
| NF2 | chr22 | 29602742 | 29604338 |
| NFKB2 | chr10 | 102392330 | 102393532 |
| NFKB2 | chr10 | 102393530 | 102396384 |
| NTRK3 | chr15 | 88255959 | 88258507 |
| NUMA1 | chr11 | 72079694 | 72081457 |
| NUMA1 | chr11 | 72081144 | 72082474 |
| OPTN | chr10 | 13098305 | 13098972 |
| OPTN | chr10 | 13098725 | 13099586 |
| OPTN | chr10 | 13099140 | 13100862 |
| PALB2 | chr16 | 23640586 | 23642296 |
| PALB2 | chr16 | 23641911 | 23642729 |
| PALB2 | chr16 | 23642511 | 23643136 |
| PAX5 | chr9 | 37033700 | 37034332 |
| PAX5 | chr9 | 37033867 | 37036186 |
| PBRM1 | chr3 | 52685073 | 52687619 |
| PDCD1LG2 | chr9 | 5508767 | 5510318 |
| PDCD1LG2 | chr9 | 5510022 | 5511326 |
| PER1 | chr17 | 8151655 | 8154138 |
| PGR | chr11 | 101128283 | 101131594 |
| PHF6 | chrX | 134371545 | 134373008 |
| PHF6 | chrX | 134372510 | 134374088 |
| PLAG1 | chr8 | 56210565 | 56211192 |
| PLAG1 | chr8 | 56210705 | 56213083 |
| PML | chr15 | 73992892 | 73995450 |
| PMS1 | chr2 | 189782314 | 189784852 |
| PMS2 | chr7 | 6008359 | 6009943 |
| PMS2 | chr7 | 6009879 | 6010698 |
| PRDM1 | chr6 | 106084549 | 106086870 |
| PRDM1 | chr6 | 106086414 | 106087095 |
| PRDM1 | chr6 | 106097085 | 106099639 |
| PRDM16 | chr1 | 3067397 | 3069953 |
| PRDM2 | chr1 | 13698619 | 13699314 |
| PRDM2 | chr1 | 13699004 | 13701008 |
| PRDM2 | chr1 | 13703076 | 13705640 |
| PRDM2 | chr1 | 13747601 | 13750165 |
| PREX2 | chr8 | 67950587 | 67953151 |
| PRKAR1A | chr17 | 68510010 | 68513161 |
| PRKAR1A | chr17 | 68513615 | 68516167 |
| PRKDC | chr8 | 47959432 | 47960770 |
| PRKDC | chr8 | 47960277 | 47961968 |
| PRLR | chr5 | 35117343 | 35118573 |
| PRLR | chr5 | 35118078 | 35119051 |
| PRLR | chr5 | 35118811 | 35119633 |
| PRLR | chr5 | 35119150 | 35119891 |
| PRLR | chr5 | 35229810 | 35231626 |
| PRLR | chr5 | 35231440 | 35232364 |
| PTCH1 | chr9 | 95506421 | 95510334 |
| PTCH1 | chr9 | 95516188 | 95518729 |
| PTEN | chr10 | 87861658 | 87864222 |
| PTGS2 | chr1 | 186679655 | 186681948 |
| PTGS2 | chr1 | 186681490 | 186682158 |
| PTPN6 | chr12 | 6944805 | 6947354 |
| PTPN6 | chr12 | 6949493 | 6950064 |
| PTPN6 | chr12 | 6949793 | 6952042 |
| PTPRD | chr9 | 10611970 | 10614492 |
| PTPRD | chr9 | 8733168 | 8735379 |
| PYCARD | chr16 | 31202004 | 31204019 |
| PYCARD | chr16 | 31203561 | 31204278 |
| RAB40AL | chrX | 102935495 | 102938036 |
| RABEP1 | chr17 | 5280675 | 5281488 |
| RABEP1 | chr17 | 5281240 | 5283045 |
| RAD51B | chr14 | 67818348 | 67819230 |
| RAD51B | chr14 | 67818878 | 67819578 |
| RAD51B | chr14 | 67819283 | 67820567 |
| RAD51C | chr17 | 58690870 | 58691700 |
| RAD51C | chr17 | 58691423 | 58693358 |
| RAD51D | chr17 | 35119093 | 35120597 |
| RAD51D | chr17 | 35120187 | 35120781 |
| RAD51D | chr17 | 35120352 | 35121650 |
| RANBP17 | chr5 | 170860116 | 170862666 |
| RAP1GDS1 | chr4 | 98259596 | 98262154 |
| RASSF1 | chr3 | 50336688 | 50338561 |
| RASSF1 | chr3 | 50338258 | 50339618 |
| RASSF1 | chr3 | 50339388 | 50340021 |
| RASSF1 | chr3 | 50340158 | 50342716 |
| RASSF5 | chr1 | 206505751 | 206508320 |
| RASSF5 | chr1 | 206555390 | 206557944 |
| RB1 | chr13 | 48301968 | 48303145 |
| RB1 | chr13 | 48302718 | 48304531 |
| RBBP8 | chr18 | 22931553 | 22934658 |
| RBM15 | chr1 | 110337544 | 110340091 |
| RBP1 | chr3 | 139539049 | 139540396 |
| RBP1 | chr3 | 139540159 | 139541610 |
| RHOH | chr4 | 40189233 | 40189883 |
| RHOH | chr4 | 40189675 | 40193724 |
| RHOH | chr4 | 40193238 | 40194411 |
| RHOH | chr4 | 40195452 | 40196946 |
| RHOH | chr4 | 40196452 | 40197679 |
| RMI2 | chr16 | 11343673 | 11344320 |
| RMI2 | chr16 | 11344008 | 11344779 |
| RMI2 | chr16 | 11344515 | 11346230 |
| RNASEL | chr1 | 182588479 | 182590725 |
| RPTOR | chr17 | 80543047 | 80543650 |
| RPTOR | chr17 | 80543482 | 80544387 |
| RPTOR | chr17 | 80544097 | 80545590 |
| RRM1 | chr11 | 4092920 | 4093779 |
| RRM1 | chr11 | 4093640 | 4095478 |
| RUNX1 | chr21 | 34887911 | 34890475 |
| RUNX1 | chr21 | 35048521 | 35050244 |
| RUNX1 | chr21 | 35049771 | 35051082 |
| RUNX1T1 | chr8 | 92016910 | 92019438 |
| RUNX1T1 | chr8 | 92062184 | 92064725 |
| RUNX1T1 | chr8 | 92094483 | 92097440 |
| RUNX1T1 | chr8 | 92098951 | 92101458 |
| RUNX1T1 | chr8 | 92102449 | 92105016 |
| RUNX3 | chr1 | 24929502 | 24932062 |
| RUNX3 | chr1 | 24964233 | 24965550 |
| RUNX3 | chr1 | 24965061 | 24966796 |
| SARDH | chr9 | 133737577 | 133740710 |
| SARDH | chr9 | 133740542 | 133741743 |
| SBDS | chr7 | 66994827 | 66996297 |
| SBDS | chr7 | 66996032 | 66997386 |
| SDHAF2 | chr11 | 61428349 | 61430906 |
| SDHB | chr1 | 17052890 | 17054643 |
| SDHB | chr1 | 17054990 | 17056451 |
| SDHC | chr1 | 161312736 | 161315161 |
| SDHD | chr11 | 112085045 | 112087595 |
| SEPT9 | chr17 | 77279635 | 77282189 |
| SEPT9 | chr17 | 77286110 | 77288676 |
| SEPT9 | chr17 | 77317868 | 77320287 |
| SEPT9 | chr17 | 77371410 | 77373966 |
| SEPT9 | chr17 | 77374310 | 77376869 |
| SEPT9 | chr17 | 77403295 | 77404584 |
| SEPT9 | chr17 | 77404270 | 77405850 |
| SEPT9 | chr17 | 77448785 | 77452008 |
| SEPT9 | chr17 | 77452214 | 77453595 |
| SEPT9 | chr17 | 77453360 | 77454768 |
| SEPT9 | chr17 | 77473464 | 77475156 |
| SEPT9 | chr17 | 77474754 | 77476017 |
| SETD2 | chr3 | 47163197 | 47164261 |
| SETD2 | chr3 | 47163767 | 47165491 |
| SFPQ | chr1 | 35192362 | 35194177 |
| SFPQ | chr1 | 35194207 | 35194835 |
| SFRP1 | chr8 | 41308694 | 41310088 |
| SFRP1 | chr8 | 41309819 | 41311253 |
| SFRP2 | chr4 | 153788307 | 153789772 |
| SFRP2 | chr4 | 153789287 | 153790859 |
| SFRP5 | chr10 | 97771221 | 97773761 |
| SLC5A8 | chr12 | 101209464 | 101211601 |
| SLC5A8 | chr12 | 101211102 | 101212025 |
| SLX4 | chr16 | 3610807 | 3612524 |
| SLX4 | chr16 | 3612397 | 3613367 |
| SMAD2 | chr18 | 47929824 | 47932918 |
| SMAD3 | chr15 | 67064079 | 67066635 |
| SMAD3 | chr15 | 67124024 | 67126502 |
| SMAD3 | chr15 | 67136359 | 67138794 |
| SMAD3 | chr15 | 67164374 | 67166939 |
| SMAD4 | chr18 | 51028436 | 51030995 |
| SMARCA4 | chr19 | 10959421 | 10960144 |
| SMARCA4 | chr19 | 10959976 | 10961919 |
| SMARCA4 | chr19 | 10982520 | 10983694 |
| SMARCA4 | chr19 | 10983460 | 10984932 |
| SMARCB1 | chr22 | 23785229 | 23785930 |
| SMARCB1 | chr22 | 23785939 | 23787746 |
| SNCG | chr10 | 86956764 | 86959311 |
| SOCS1 | chr16 | 11255404 | 11257960 |
| SOCS3 | chr17 | 78359306 | 78361858 |
| SPECC1 | chr17 | 20007566 | 20010116 |
| SPECC1 | chr17 | 20085241 | 20087803 |
| SPECC1 | chr17 | 20154216 | 20156771 |
| SPEN | chr1 | 15846361 | 15847151 |
| SPEN | chr1 | 15846836 | 15848643 |
| SRGAP3 | chr3 | 9248907 | 9251469 |
| STK11 | chr19 | 1204018 | 1205006 |
| STK11 | chr19 | 1204733 | 1206579 |
| SUFU | chr10 | 102502184 | 102504737 |
| SYK | chr9 | 90799910 | 90800754 |
| SYK | chr9 | 90800335 | 90800974 |
| SYK | chr9 | 90800519 | 90802707 |
| SYK | chr9 | 90825639 | 90828200 |
| TCEA1 | chr8 | 54021680 | 54023017 |
| TCEA1 | chr8 | 54023080 | 54024063 |
| TET1 | chr10 | 68558579 | 68559299 |
| TET1 | chr10 | 68559049 | 68561136 |
| TFAP2A | chr6 | 10411605 | 10414121 |
| TFAP2A | chr6 | 10414460 | 10417017 |
| TFAP2A | chr6 | 10418793 | 10421339 |
| TFG | chr3 | 100707511 | 100708546 |
| TFG | chr3 | 100708131 | 100710328 |
| TGFBR2 | chr3 | 30604732 | 30607277 |
| THBS1 | chr15 | 39579298 | 39579871 |
| THBS1 | chr15 | 39579373 | 39581859 |
| THRAP3 | chr1 | 36222891 | 36225195 |
| TIMP3 | chr22 | 32799042 | 32801452 |
| TIMP3 | chr22 | 32800957 | 32801585 |
| TLX3 | chr5 | 171307515 | 171310066 |
| TMEFF2 | chr2 | 192194139 | 192196692 |
| TMEM127 | chr2 | 96265234 | 96267155 |
| TMEM127 | chr2 | 96266899 | 96267791 |
| TNFAIP3 | chr6 | 137865409 | 137867971 |
| TOP2A | chr17 | 40417181 | 40418825 |
| TOP2A | chr17 | 40418546 | 40419401 |
| TP53 | chr17 | 7674714 | 7675643 |
| TP53 | chr17 | 7675394 | 7677192 |
| TP53 | chr17 | 7686773 | 7689328 |
| TP63 | chr3 | 189629648 | 189632210 |
| TP63 | chr3 | 189787884 | 189789190 |
| TP63 | chr3 | 189788707 | 189789697 |
| TP63 | chr3 | 189789222 | 189790268 |
| TP63 | chr3 | 189789769 | 189790448 |
| TP73 | chr1 | 3650789 | 3653346 |
| TP73 | chr1 | 3688903 | 3691452 |
| TP73 | chr1 | 3696269 | 3698820 |
| TRIM33 | chr1 | 114510383 | 114511920 |
| TRIM33 | chr1 | 114511426 | 114512336 |
| TRIM33 | chr1 | 114512083 | 114512935 |
| TSC1 | chr9 | 132943865 | 132946411 |
| TSC2 | chr16 | 2046209 | 2046953 |
| TSC2 | chr16 | 2046699 | 2048772 |
| TSHR | chr14 | 80953999 | 80956314 |
| TTL | chr2 | 112480389 | 112482926 |
| TUBB3 | chr16 | 89920242 | 89924058 |
| VDR | chr12 | 47904252 | 47906810 |
| VHL | chr3 | 10140079 | 10140808 |
| VHL | chr3 | 10140576 | 10141539 |
| VHL | chr3 | 10141314 | 10142417 |
| WIF1 | chr12 | 65120797 | 65122592 |
| WIF1 | chr12 | 65122320 | 65123022 |
| WIF1 | chr12 | 65122524 | 65123329 |
| WRN | chr8 | 31031483 | 31034043 |
| XPA | chr9 | 97696640 | 97698772 |
| XPA | chr9 | 97698585 | 97699193 |
| XPC | chr3 | 14177892 | 14180443 |
| YWHAE | chr17 | 1399492 | 1400886 |
| YWHAE | chr17 | 1400601 | 1401278 |
| YWHAE | chr17 | 1401182 | 1402031 |
| ZBTB16 | chr11 | 114057938 | 114061344 |
| ZMYM2 | chr13 | 19956899 | 19959441 |
| ZNF331 | chr19 | 53519143 | 53520292 |
| ZNF331 | chr19 | 53520033 | 53521588 |
| ZNF331 | chr19 | 53521098 | 53521696 |
| ZNF331 | chr19 | 53536663 | 53539569 |
| ZNF331 | chr19 | 53552868 | 53553470 |
| ZNF331 | chr19 | 53553048 | 53556087 |
| ZNF668 | chr16 | 31064314 | 31065859 |
| ZNF668 | chr16 | 31065546 | 31066733 |
| ZNF668 | chr16 | 31072734 | 31075544 |
| ZNF668 | chr16 | 31075319 | 31076073 |
| ZRSR2 | chrX | 15788680 | 15789399 |
| ZRSR2 | chrX | 15789350 | 15791219 |
