# Supplementary material for: Assessment of tumor suppressor promoter methylation in healthy individuals
Source: Clin Epigenetics. 2020 Aug 28;12:131. doi: 10.1186/s13148-020-00920-7 (PMC7455917; doi:10.1186/s13148-020-00920-7)
Supplement: Supplementary file 5 — Additional file 5: Supplementary Table S3. Genes with >99 confidence level difference in methylation ratio between a minority (one third or less) of samples versus the majority. [file 13148_2020_920_MOESM5_ESM.docx]

**Supplementary Table S3**: Genes with >99 confidence level difference in methylation ratio between a minority (one third or less) of samples versus the majority.

| **Gene Name** | **Gene Capture Region** | **Min. Methylation** | **Max. Methylation** | **Diff. Methylation** |
| --- | --- | --- | --- | --- |
| GAS7 | chr17:10199716-10200316 | 0.267 | 0.9332 | 0.6662 |
| ELAC2 | chr17:13019069-13019845 | 0.458125 | 0.8331875 | 0.3750625 |
| GSTM1 | chr1:109686327-109687046 | 0.64378571 | 1 | 0.35621429 |
| THBS1 | chr15-39579298-39579871 | 0.46709091 | 0.78852381 | 0.3214329 |
| CIITA | chr16:10874982-10875928 | 0.25111111 | 0.55766667 | 0.30655556 |
| RASSF1 | chr3:50339388-50340021 | 0.178625 | 0.472 | 0.293375 |
| CHN1 | chr2:174846842-174848034 | 0.214125 | 0.5074 | 0.293275 |
| MSH2 | chr2:47401613-47402319 | 0.58965079 | 0.87342105 | 0.28377026 |
| PALB2 | chr16:23642511-23643136 | 0.63333333 | 0.91338462 | 0.28005128 |
| RUNX3 | chr1:24964233-24965550 | 0.39197917 | 0.64785714 | 0.25587798 |
| TP63 | chr3:189789769-189790448 | 0.66125 | 0.905875 | 0.244625 |
| PDCD1LG2 | chr9:5510022-5511326 | 0.31816667 | 0.55114286 | 0.23297619 |
| AIP | chr11:67481632-67482276 | 0.67157143 | 0.9002 | 0.22862857 |
| GPC3 | chrX:133986729-133987434 | 0.58417647 | 0.80361905 | 0.21944258 |
| AIP | chr11:67482202-67482880 | 0.10346154 | 0.32140909 | 0.21794755 |
| GSTP1 | chr11:67581895-67582976 | 0.26728 | 0.48343478 | 0.21615478 |
| AIP | chr11:67481257-67481869 | 0.78566667 | 1 | 0.21433333 |
| XPA | chr9:97698585-97699193 | 0.6991 | 0.913 | 0.2139 |
| APC | chr5:112736082-112736959 | 0.63608696 | 0.84789474 | 0.21180778 |
| CTCFL | chr20:57524096-57527440 | 0.65539623 | 0.86627679 | 0.21088056 |
| CASP8 | chr2:201259179-201260169 | 0.36975 | 0.5799 | 0.21015 |
| ZNF668 | chr16:31064314-31065859 | 0.45134426 | 0.65835088 | 0.20700661 |
| COX6C | chr8:99895019-99895783 | 0.7228 | 0.9216 | 0.1988 |
| ERCC3 | chr2:127294647-127295832 | 0.530875 | 0.72734884 | 0.19647384 |
| CLDN3 | chr7:73771154-73772013 | 0.67383333 | 0.86988889 | 0.19605556 |
| AXIN2 | chr17:65562733-65563403 | 0.743 | 0.9122 | 0.1692 |
| PRLR | chr5:35118811-35119633 | 0.63226667 | 0.7974375 | 0.16517083 |
| GAS7 | chr17:9958680-9960456 | 0.33153571 | 0.49331461 | 0.16177889 |
| MSH6 | chr2:47781508-47782351 | 0.64046939 | 0.80116667 | 0.16069728 |
| CNBP | chr3:129184602-129185353 | 0.4559 | 0.61326087 | 0.15736087 |
| RMI2 | chr16:11343673-11344320 | 0.81330769 | 0.96728571 | 0.15397802 |
| CDKN1A | chr6:36675451-36676113 | 0.74827778 | 0.90173077 | 0.15345299 |
| DAPK1 | chr9:87495448-87496206 | 0.7115 | 0.8614 | 0.1499 |
| ERCC2 | chr19:45371478-45372154 | 0.78905556 | 0.93785 | 0.14879444 |
| EMP3 | chr19:48323926-48324869 | 0.69804545 | 0.84676667 | 0.14872121 |
| ZRSR2 | chrX:15788680-15789399 | 0.77790909 | 0.92266667 | 0.14475758 |
| GAS7 | chr17:10035978-10038207 | 0.53445833 | 0.67918 | 0.14472167 |
| MEN1 | chr11:64812298-64813077 | 0.79070588 | 0.93247619 | 0.14177031 |
| LMNA | chr1:156124336-156125702 | 0.56943243 | 0.70513158 | 0.13569915 |
| VHL | chr3:10140079-10140808 | 0.808 | 0.94353846 | 0.13553846 |
| FOXO4 | chrX:71094692-71096928 | 0.12563905 | 0.25917514 | 0.13353609 |
| PRKAR1A | chr17:68513615-68516167 | 0.61084906 | 0.7437963 | 0.13294724 |
| YWHAE | chr17:1401182-1402031 | 0.73731034 | 0.87015625 | 0.13284591 |
| ZNF331 | chr19:53536663-53539569 | 0.38020702 | 0.51064111 | 0.1304341 |
| EMP3 | chr19:48324401-48325017 | 0.68806897 | 0.81502778 | 0.12695881 |
| CDKN2D | chr19:10569864-10570540 | 0.747 | 0.87295455 | 0.12595455 |
| HERPUD1 | chr16:56930313-56931125 | 0.79944828 | 0.92415152 | 0.12470324 |
| GSTM1 | chr1:109686097-109686745 | 0.804125 | 0.92828571 | 0.12416071 |
| CHEK1 | chr11:125623875-125624579 | 0.76829412 | 0.8918 | 0.12350588 |
| CDKN1A | chr6:36674681-36675697 | 0.79684615 | 0.91885294 | 0.12200679 |
| SPECC1 | chr17:20085241-20087803 | 0.73038983 | 0.85217857 | 0.12178874 |
| RASSF1 | chr3:50338258-50339618 | 0.09756364 | 0.21778 | 0.12021636 |
| DICER1 | chr14:95136031-95136667 | 0.77166667 | 0.89066667 | 0.119 |
| DICER1 | chr14:95158704-95159791 | 0.69188889 | 0.80955814 | 0.11766925 |
| EMP3 | chr19:48324661-48326149 | 0.21446667 | 0.33020513 | 0.11573846 |
| SARDH | chr9:133740542-133741743 | 0.76378378 | 0.87497872 | 0.11119494 |
| ATR | chr3:142579817-142580609 | 0.83028571 | 0.94142857 | 0.11114286 |
| ZNF668 | chr16:31065546-31066733 | 0.55264407 | 0.66275472 | 0.11011065 |
| WIF1 | chr12:65122320-65123022 | 0.801225 | 0.91025581 | 0.10903081 |
| SYK | chr9:90825639-90828200 | 0.3525 | 0.45986486 | 0.10736486 |
| PRDM2 | chr1:13698619-13699314 | 0.83507692 | 0.94130769 | 0.10623077 |
| SARDH | chr9:133737577-133740710 | 0.47562143 | 0.58176129 | 0.10613986 |
| CASP8 | chr2:201256864-201257468 | 0.65068966 | 0.75616129 | 0.10547164 |
| HERPUD1 | chr16:56930633-56931495 | 0.74575 | 0.850725 | 0.104975 |
| ESR1 | chr6:151803894-151806772 | 0.74583582 | 0.85061111 | 0.10477529 |
| FBXO11 | chr2:47887940-47890324 | 0.75364706 | 0.85766667 | 0.10401961 |
| FBXW7 | chr4:152352181-152354741 | 0.77510638 | 0.87840426 | 0.10329787 |
| MSH2 | chr2:47401288-47401885 | 0.81046154 | 0.91293939 | 0.10247786 |
| BUB1B | chr15:40159231-40160455 | 0.8190625 | 0.92145 | 0.1023875 |
| MEN1 | chr11:64811758-64812619 | 0.82664706 | 0.92855 | 0.10190294 |
| TP63 | chr3:189788707-189789697 | 0.75686957 | 0.85870833 | 0.10183877 |
| RAD51C | chr17:58691423-58693358 | 0.30288344 | 0.40332738 | 0.10044395 |
| GPC3 | chrX:133984871-133986973 | 0.29245887 | 0.38831837 | 0.09585949 |
| PRLR | chr5:35229810-35231626 | 0.14794595 | 0.2425 | 0.09455405 |
| KEAP1 | chr19:10504033-10504794 | 0.82555814 | 0.92007273 | 0.09451459 |
| GMPS | chr3:155868983-155869898 | 0.73588235 | 0.82821429 | 0.09233193 |
| CDK12 | chr17:39460362-39461006 | 0.81951064 | 0.91105556 | 0.09154492 |
| IL21R | chr16:27425579-27427633 | 0.54186667 | 0.6312 | 0.08933333 |
| DDX53 | chrX:22998633-23000742 | 0.73484211 | 0.82374359 | 0.08890148 |
| DICER1 | chr14:95136276-95137349 | 0.80775 | 0.89625 | 0.0885 |
| GATA1 | chrX:48784803-48787354 | 0.69413115 | 0.78036667 | 0.08623552 |
| RHOH | chr4:40196452-40197679 | 0.1059375 | 0.19139583 | 0.08545833 |
| GAS7 | chr17:10025528-10028002 | 0.75524638 | 0.83964286 | 0.08439648 |
| FANCD2 | chr3:10024958-10025701 | 0.740625 | 0.82478947 | 0.08416447 |
| TCEA1 | chr8:54023080-54024063 | 0.71917647 | 0.80277778 | 0.08360131 |
| FHL1 | chrX:136144923-136149352 | 0.28371208 | 0.36647967 | 0.08276759 |
| MTUS2 | chr13:29022834-29025397 | 0.77007692 | 0.8517963 | 0.08171937 |
| ZNF331 | chr19:53519143-53520292 | 0.84966667 | 0.92520513 | 0.07553846 |
| RAB40AL | chrX:102935495-102938036 | 0.7445 | 0.81769565 | 0.07319565 |
| FANCI | chr15:89242185-89244752 | 0.12242857 | 0.19496651 | 0.07253794 |
| AIP | chr11:67482382-67483805 | 0.05165517 | 0.12285333 | 0.07119816 |
| NF1 | chr17:31093597-31095703 | 0.1122967 | 0.18223936 | 0.06994266 |
| TET1 | chr10:68558579-68559299 | 0.85383333 | 0.92327778 | 0.06944444 |
| CREB3L1 | chr11:46276972-46278418 | 0.09306164 | 0.15993671 | 0.06687507 |
| RPTOR | chr17:80544097-80545590 | 0.1459661 | 0.2102 | 0.0642339 |
| CIITA | chr16:10876007-10878457 | 0.12363333 | 0.18701667 | 0.06338333 |
| RABEP1 | chr17:5281240-5283045 | 0.04147951 | 0.10329317 | 0.06181366 |
| MLF1 | chr3:158569396-158571932 | 0.11071212 | 0.17092366 | 0.06021154 |
| SMAD3 | chr15:67164374-67166939 | 0.84651145 | 0.90663636 | 0.06012491 |
| EPHB6 | chr7:142861293-142863543 | 0.8228 | 0.882 | 0.0592 |
| SMAD3 | chr15:67136359-67138794 | 0.80484615 | 0.85901818 | 0.05417203 |
| MTUS2 | chr13:29426863-29429423 | 0.12653846 | 0.18018478 | 0.05364632 |
| ZNF331 | chr19:53521098-53521696 | 0.00722727 | 0.06021277 | 0.05298549 |
| ERG | chr21:38661006-38663557 | 0.12055414 | 0.17197436 | 0.05142022 |
| PAX5 | chr9:37033867-37036186 | 0.03284109 | 0.0838677 | 0.05102662 |
| PER1 | chr17:8151655-8154138 | 0.18613609 | 0.23451765 | 0.04838155 |
| PRDM2 | chr1:13699004-13701008 | 0.13710309 | 0.18511465 | 0.04801156 |
| TRIM33 | chr1:114511426-114512336 | 0.01061728 | 0.0584382 | 0.04782092 |
| PTPRD | chr9:10611970-10614492 | 0.03786555 | 0.08445643 | 0.04659089 |
| ZRSR2 | chrX:15789350-15791219 | 0.05594595 | 0.10211392 | 0.04616798 |
| MIR127 | chr14:100881198-100883763 | 0.863996 | 0.91005426 | 0.04605826 |
| DAPK1 | chr9:87496675-87499315 | 0.06479781 | 0.11080585 | 0.04600804 |
| WIF1 | chr12:65122524-65123329 | 0.85470833 | 0.90033333 | 0.045625 |
| RRM1 | chr11:4093640-4095478 | 0.06654438 | 0.11207182 | 0.04552744 |
| RUNX1T1 | chr8:92102449-92105016 | 0.05581071 | 0.10084099 | 0.04503028 |
| DNMT3A | chr2:25251544-25254071 | 0.23771138 | 0.28177824 | 0.04406686 |
| PTGS2 | chr1:186679655-186681948 | 0.02255769 | 0.06597436 | 0.04341667 |
| LTBP2 | chr14:74611551-74613167 | 0.01825564 | 0.05976573 | 0.0415101 |
| SDHC | chr1:161312736-161315161 | 0.17800613 | 0.21936747 | 0.04136133 |
| RNASEL | chr1:182588479-182590725 | 0.29172414 | 0.33281667 | 0.04109253 |
| KMT2C | chr7:152436898-152437784 | 0.13988889 | 0.17907407 | 0.03918519 |
| THBS1 | chr15:39579373-39581859 | 0.06697417 | 0.10594161 | 0.03896744 |
| APC | chr5:112705736-112708295 | 0.12996644 | 0.16828276 | 0.03831632 |
| SYK | chr9:90800519-90802707 | 0.02826238 | 0.06411881 | 0.03585644 |
| FANCG | chr9:35080079-35080717 | 0.00025806 | 0.03545 | 0.03519194 |
| ERCC5 | chr13:102844882-102845734 | 0.03097674 | 0.06593023 | 0.03495349 |
| HOXA10 | chr7:27173559-27176123 | 0.18291319 | 0.2160102 | 0.03309701 |
| CDKN2D | chr19:10568175-10570125 | 0.03989744 | 0.07194643 | 0.03204899 |
| CDX2 | chr13:27968588-27971148 | 0.04558077 | 0.077 | 0.03141923 |
| SMARCB1 | chr22:23785939-23787746 | 0.08094545 | 0.11188496 | 0.0309395 |
| CHFR | chr12:132886841-132889287 | 0.0870375 | 0.11648447 | 0.02944697 |
| BMP3 | chr4:81030219-81031752 | 0.00888073 | 0.03826728 | 0.02938655 |
| COX6C | chr8:99893234-99895453 | 0.1317 | 0.159575 | 0.027875 |
| RASSF1 | chr3:50340158-50342716 | 0.11970815 | 0.14754274 | 0.02783458 |
| YWHAE | chr17:1399492-1400886 | 0.02367876 | 0.05074479 | 0.02706604 |
| IGFBP3 | chr7:45920494-45923044 | 0.01698011 | 0.04373239 | 0.02675228 |
| ESR1 | chr6:151806314-151808440 | 0.07603483 | 0.10145055 | 0.02541572 |
| STK11 | chr19:1204733-1206579 | 0.05956851 | 0.08385359 | 0.02428508 |
| CTNNB1 | chr3:41197680-41200220 | 0.04452632 | 0.06772425 | 0.02319794 |
| ASXL1 | chr20:32356589-32359117 | 0.02076103 | 0.04381522 | 0.02305419 |
| CDC73 | chr1:193120186-193122737 | 0.11585238 | 0.13876526 | 0.02291288 |
| LRP5 | chr11:68310830-68313385 | 0.0979354 | 0.11970466 | 0.02176926 |
| CDKN2A | chr9:21974060-21976361 | 0.01971134 | 0.04114063 | 0.02142928 |
| TFAP2A | chr6:10411605-10414121 | 0.01627778 | 0.03769811 | 0.02142034 |
| LMNA | chr1:156080779-156083321 | 0.0345 | 0.05534583 | 0.02084583 |
| KDM5C | chrX:53224644-53227201 | 0.08681395 | 0.10762857 | 0.02081462 |
| TCEA1 | chr8:54021680-54023017 | 0.00110286 | 0.02183429 | 0.02073143 |
| FUS | chr16:31178329-31180898 | 0.0731875 | 0.09373226 | 0.02054476 |
| TSC1 | chr9:132943865-132946411 | 0.13645815 | 0.15683857 | 0.02038042 |
| GAS7 | chr17:10197776-10199919 | 0.04043391 | 0.06065833 | 0.02022443 |
| SOCS3 | chr17:78359306-78361858 | 0.05231161 | 0.07126316 | 0.01895154 |
| PRDM1 | chr6:106086414-106087095 | 0.00520408 | 0.02410204 | 0.01889796 |
| FANCD2 | chr3:10025203-10027217 | 0.07266667 | 0.09154762 | 0.01888095 |
| SFRP5 | chr10:97771221-97773761 | 0.05484483 | 0.07320339 | 0.01835856 |
| DNMT3A | chr2:25341140-25344370 | 0.13111727 | 0.14939663 | 0.01827936 |
| TFG | chr3:100708131-100710328 | 0.09369626 | 0.11111111 | 0.01741485 |
| PML | chr15:73992892-73995450 | 0.1777931 | 0.19518421 | 0.01739111 |
| NTRK3 | chr15:88255959-88258507 | 0.01381505 | 0.03112883 | 0.01731379 |
| FAM46C | chr1:117604214-117606766 | 0.0723431 | 0.08963968 | 0.01729658 |
| SPECC1 | chr17:20154216-20156771 | 0.0927191 | 0.10973408 | 0.01701498 |
| CHEK2 | chr22:28741055-28743614 | 0.0707439 | 0.08716463 | 0.01642073 |
| TMEFF2 | chr2:192194139-192196692 | 0.00902866 | 0.0251465 | 0.01611783 |
| RASSF5 | chr1:206505751-206508320 | 0.06414563 | 0.0801873 | 0.01604167 |
| HIC1 | chr17:2053320-2057100 | 0.01040088 | 0.0256769 | 0.01527602 |
| NDRG1 | chr8:133296524-133299086 | 0.03089781 | 0.04553731 | 0.0146395 |
| MNX1 | chr7:157008657-157012437 | 0.01151988 | 0.02576328 | 0.0142434 |
| CHN1 | chr2:175004476-175007018 | 0.02566148 | 0.03965234 | 0.01399087 |
| RUNX1 | chr21:35048521-35050244 | 0.00495122 | 0.01887805 | 0.01392683 |
| FANCE | chr6:35450583-35453141 | 0.06306047 | 0.0769 | 0.01383953 |
| KEAP1 | chr19:10502027-10503898 | 0.00210759 | 0.01563354 | 0.01352595 |
| KLF6 | chr10:3784501-3787050 | 0.07115254 | 0.08447059 | 0.01331805 |
| EIF4A2 | chr3:186781792-186784353 | 0.05571111 | 0.06895203 | 0.01324092 |
| TSC2 | chr16:2046699-2048772 | 0.0302377 | 0.04260729 | 0.01236958 |
| FLCN | chr17:17236411-17238116 | 0.00121739 | 0.01342623 | 0.01220884 |
| MLLT11 | chr1:151057906-151060456 | 0.00941212 | 0.02024699 | 0.01083487 |
| TTL | chr2:112480389-112482926 | 0.01142742 | 0.02198433 | 0.01055691 |
| SOCS1 | chr16:11255404-11257960 | 0.02542857 | 0.03473219 | 0.00930362 |
| ERCC5 | chr13:102845327-102846619 | 0.00186395 | 0.01059603 | 0.00873208 |
| RAP1GDS1 | chr4:98259596-98262154 | 0.00961538 | 0.01833149 | 0.00871611 |
| FANCG | chr9:35079240-35080574= | 0.00106748 | 0.00960625 | 0.00853877 |
| GSTP1 | chr11:67583140-67584374 | 0.00111189 | 0.00945185 | 0.00833996 |
| GATA3 | chr10:8052925-8055161 | 0.00667857 | 0.01418375 | 0.00750517 |
| CIC | chr19:42282884-42285452 | 0.00665915 | 0.01394798 | 0.00728883 |
| KDM6A | chrX:44871397-44873963 | 0.01372443 | 0.02056825 | 0.00684381 |
| PTCH1 | chr9:95506421-95510334 | 0.00300896 | 0.00970567 | 0.00669671 |
| CNBP | chr3:129183187-129185101 | 0.01750847 | 0.02404721 | 0.00653874 |
| ELAC2 | chr17:13017290-13018788 | 0.00215842 | 0.00842079 | 0.00626238 |
| FOXO1 | chr13:40665821-40668369 | 0.00181074 | 0.00801285 | 0.00620211 |
| ARHGEF12 | chr11:120335132-120337693 | 0.00480966 | 0.01064873 | 0.00583907 |
| PMS2 | chr7:6008359-6009943 | 0.00144554 | 0.00699507 | 0.00554953 |
| PLAG1 | chr8:56210705-56213083 | 0.0019116 | 0.00701366 | 0.00510206 |
| OPTN | chr10:13099140-13100862 | 0.00357592 | 0.00810825 | 0.00453233 |
| PRKDC | chr8:47959432-47960770 | 0.00083974 | 0.00531098 | 0.00447123 |
| KMT2C | chr7:152435227-152437363 | 0.00568357 | 0.00990611 | 0.00422254 |
| IKZF1 | chr7:50304733-50305451 | 0.00033803 | 0.00414667 | 0.00380864 |
| BCL7A | chr12:122021689-122022739 | 0.00071053 | 0.00442164 | 0.00371112 |
| ARID1B | chr6:156778004-156778717 | 0.00054167 | 0.0041157 | 0.00357404 |
| FBXO11 | chr2:47905020-47907557 | 0.00098913 | 0.00387126 | 0.00288213 |
| SETD2 | chr3:47163767-47165491 | 0.00089568 | 0.00375618 | 0.0028605 |
| FANCM | chr14:45134154-45136711 | 0.00106667 | 0.00374661 | 0.00267994 |
| ARID2 | chr12:45728058-45729857 | 0.00073929 | 0.00308664 | 0.00234736 |
| SUFU | chr10:102502184-102504737 | 0.0010383 | 0.00314346 | 0.00210516 |
| RUNX1 | chr21:34887911-34890475 | 0.00093905 | 0.00295437 | 0.00201533 |
| SETD2 | chr3:47163197-47164261 | 0.00088115 | 0.00256767 | 0.00168652 |
| TRIM33 | chr1:114510383-114511920 | 0.00104319 | 0.00269767 | 0.00165449 |
| RBM15 | chr1:110337544-110340091 | 0.00123099 | 0.00285429 | 0.0016233 |
